# Reassessing the Heterogeneous Effects of Greenspace on Air Pollution Conditioned on Seasons, Vegetation Types, and Street Structures

**DOI:** 10.1021/acs.est.5c15520

**Published:** 2026-05-27

**Authors:** Zhenchuan Yang, Mei-Po Kwan, Yan Zhang

**Affiliations:** † Institute of Space and Earth Information Science, 26451 The Chinese University of Hong Kong,Shatin, Hong Kong Special Administrative Region, China; ‡ Department of Geography and Resource Management, 26451 The Chinese University of Hong Kong,Shatin,Hong Kong Special Administrative Region, China

**Keywords:** greenspace, air pollution, seasons, vegetation types, street structures, double machine learning

## Abstract

Urban greenspace is generally assumed to mitigate air pollution. However, clear large-scale real-world evidence remains limited, particularly regarding how its effects vary with seasons, vegetation types, and street structures. To bridge this gap, this study integrates 4.254 million historical street view images, 1.894 million records of mobile monitoring air pollution data, and three-dimensional urban morphology data in Hong Kong, employing a double machine learning (DML) framework to disentangle the complex effects of urban greenspace. The results show that the DML framework provides more robustness and less biased estimates than conventional models. The estimated greenspace’s effects vary substantially across vegetation types and seasons: grass reduced PM_2.5_ levels in spring (θ = −8.464) and autumn (θ = −6.506); trees increased PM_2.5_ levels in summer (θ = 0.762); and mid-level vegetation showed opposite effects in spring (θ = −2.275) and winter (θ = 3.480). Mechanistically, these differences reflect the relative magnitudes of biogenic volatile organic compounds (BVOC) emissions, deposition processes, and aerodynamic effects. Moreover, the street height-to-width ratio significantly moderates greenspace’s effects due to the ventilation alteration within street canyons. These insights advance current knowledge and offer an evidence base for policy-making in urban greenspace construction and renewal and air pollution control.

## Graphical abstract

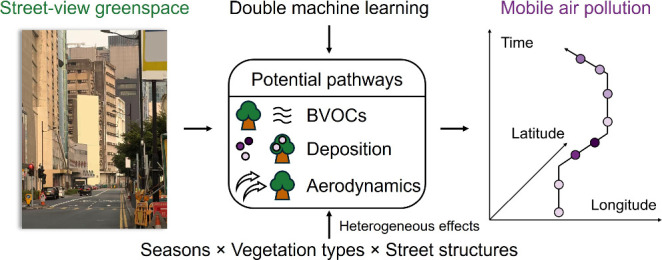

## 1 Introduction

Air pollution is a major global threat to human health.^1^ Meanwhile, urban greenspace is widely assumed to mitigate air pollution.^2,3^ Previous research evidence shows that vegetation can directly capture airborne pollutants (e.g., particulate matter)^4^ and reduce air pollution by lowering building energy consumption due to its mitigating effect on the urban heat island effect.^5^ Nonetheless, greenspace may also exacerbate air pollution by impeding ventilation^6^ and emitting biogenic volatile organic compounds (BVOCs).^7,8^ Given these complex (mitigating or exacerbating) effects of urban greenspace on air pollution, it is critical to comprehensively understand the relationship between urban greenspace and air pollution to optimize urban greenspace configuration for public health.

To explore this complexity, initially, research focused heavily on microscale analyses using experimental or model-based approaches, such as atmospheric chemistry, computational fluid dynamics (CFD), and ENVI-met models.^9–12^ These studies systematically examined the heterogeneous effects of different vegetation types across street canyons and indicated that taller vegetation is more likely to exert adverse aerodynamic effects, thereby exacerbating air pollution, while depositional effects of vegetation may become stronger under conditions of low wind speed and high pollutant concentrations. Other studies have further incorporated the effects of BVOC emissions from vegetation, suggesting that the net effect of urban greenspace on PM_2.5_ concentrations may be an increase rather than a reduction.^7^ Nevertheless, the ecological validity of these models remains questionable.^6,13,14^ Moreover, model input parameters (e.g., deposition velocity) are often uncertain and may not adequately capture the complexities of real-world urban environments. Compared with the simulation method, empirical studies in the real world could solve the problem of authenticity. Those methods can be classified into two categories. One category adopts a micro perspective and is typically conducted on one or several streets or parks within a city.^15–17^ In contrast to simulation-based studies, empirical investigations conducted in real-world environments using limited sites have generally indicated that vegetation has the potential to mitigate particulate matter pollution. However, these findings are limited by the nonstationarity of the relationships.^18,19^ Specifically, greenspace effects derived from specific sample locations may not generalize to other urban settings, potentially misguiding urban planning strategies. The second empirical approach takes a macro-perspective, encompassing the entire urban area. These investigations typically rely on two-dimensional (2D) greenspace indicators (e.g., the Normalized Difference Vegetation Index (NDVI) and land use/cover classifications) derived from satellite imagery.^20–22^ They typically observed the pollutant removal effects of vegetation mainly driven by dry deposition and concluded that urban greenspace can mitigate air pollution. However, such studies often fail to capture the intricate microcharacteristics of urban and greenspace morphology, which may in turn bias both their estimates and policy implications. Although empirical studies at both micro- and city levels are technically feasible, using methods such as photogrammetry and LiDAR to depict detailed three-dimensional (3D) urban and greenspace morphology,^23,24^ these techniques are often deemed impractical due to their substantial economic and temporal costs.

With the rapid advancement of urban sensing technologies and deep learning algorithms,^25–27^ it is now feasible to efficiently and cost-effectively examine the effects of urban greenspace on air pollution at both micro- and city levels. Street view imagery is one of the most important emerging urban sensing data sets. It could capture the three-dimensional urban greenspace and then calculate the green view index (GVI) and green view factor (GVF).^28–30^ In addition, some annotated data sets (e.g., ADE20K) and deep learning-based semantic segmentation algorithms (e.g., Mask2Former) have further enabled the isolation of vegetation types, including trees, mid-level vegetation, and grass.^31,32^ Additionally, the integration of remote sensing imagery with machine learning algorithms has facilitated the construction of large-scale microurban morphology data sets, such as the three-dimensional Global Building Footprints (3D-GloBFP) data set, which provides accurate global building boundaries and heights.^33^ The high-resolution spatiotemporal air pollution mapping has also been advanced through the combined use of static monitoring, portable sensors, crowdsourced data, remote sensing, and model simulation.^18,34–36^ Relying on these extensive spatiotemporal data sets, pilot studies have been conducted to investigate the relationships between urban greenspace and air pollution in real-world settings.^28,34^ For example, in the investigation encompassing the entire road network of Dublin City, researchers employed Google Street View-derived urban greenspace and Google Air View-derived pollution levels, finding significant negative associations between them.^34^

Despite these advances, several gaps remain. First, large-scale empirical studies have predominantly focused on overall measures of greenspace (represented by either the NDVI or GVI),^2,20,34^ failing to differentiate how various vegetation types (i.e., trees, mid-level vegetation, and grass) influence this relationship. In fact, vegetation types differ substantially in BVOC emissions, pollutant deposition, and aerodynamic effects^6,37^ and may therefore exert considerable heterogeneous effects on air pollution. Second, the impact of urban form, particularly the street canyon effect caused by street structures, on air pollution has been highlighted in small-scale^28^ or experimental studies^12,38^ but lacks systematic investigation in large-scale empirical research, especially regarding its moderating role in generating heterogeneous effects of greenspace on air pollution. Finally, the temporal dynamics of the greenspace-air pollution relationship, especially seasonality, are frequently overlooked or on a small scale.^17,39,40^ However, both greenspace and air pollution exhibit strong seasonal heterogeneity, which likely alters the effect of greenspace.^6,41–43^ For instance, urban vegetation typically exhibits stronger BVOC emissions in summer, which may promote the formation of secondary pollutants, thereby exacerbating air pollution.^44,45^ The deposition of greenspace in street canyons was found to be more effective under higher concentrations.^12^ Neglecting these three critical factors (i.e., vegetation types, street structures, and seasonality) can lead to confusing or even contradictory findings, undermining efforts to develop precise urban design and public health policies.

To address these research gaps, this study collected high-precision three-dimensional urban building data and high-quality urban sensing data in Hong Kong (including historical street-view imagery and mobile air pollution measurements). The state-of-the-art Mask2Former algorithm was employed to identify different vegetation types in the street view images. Aggregating these data to street and seasonal levels and utilizing the double machine learning (DML) method, the investigation evaluated the average and heterogeneous effects of urban green space on air pollution. Through these efforts, the study aims to answer the question: How do effects (mitigating or exacerbating) of urban greenspace on air pollution vary across seasons, vegetation types (i.e., trees, mid-level vegetation, grass), and street structures?

## 2 Materials and Methods

### 2.1 Study Area

This study was conducted in Hong Kong, a densely populated and highly urbanized city in Southeast China (Figure S1A). Figure S1B,C present two street samples, Sha Tin Center Street and New Praya in Kennedy Town. The annual mean concentrations of PM_2.5_ and PM_10_ recorded in 2022 were 14.611 and 24.277 μg/m^3^, respectively,^46^ which exceed the World Health Organization’s (WHO) air quality guidelines of 10 μg/m^3^ for PM_2.5_ and 20 μg/m^3^ for PM_10._ ^47^ Primary sources of air pollution include public electricity generation, road transport, navigation, and other combustion such as nonroad mobile machinery,^48^ indicating that most pollution occurs in urban areas. We refined the study area to the urban areas of Hong Kong, reflecting considerations of research design and data availability, which is detailed in Supplementary Text S1. Specifically, we delineated urban areas using the area dominance method^49^ and 10 m resolution impervious surface data.^50^ The refined study area is depicted in Figure 1. A detailed description of the area dominance method is provided in Supplementary Text S2, and an interpretation of the distribution of urban areas is presented in Supplementary Text S3. Furthermore, we set the analysis unit as the street in urban areas, aligning with our study objective.

**Figure 1:**
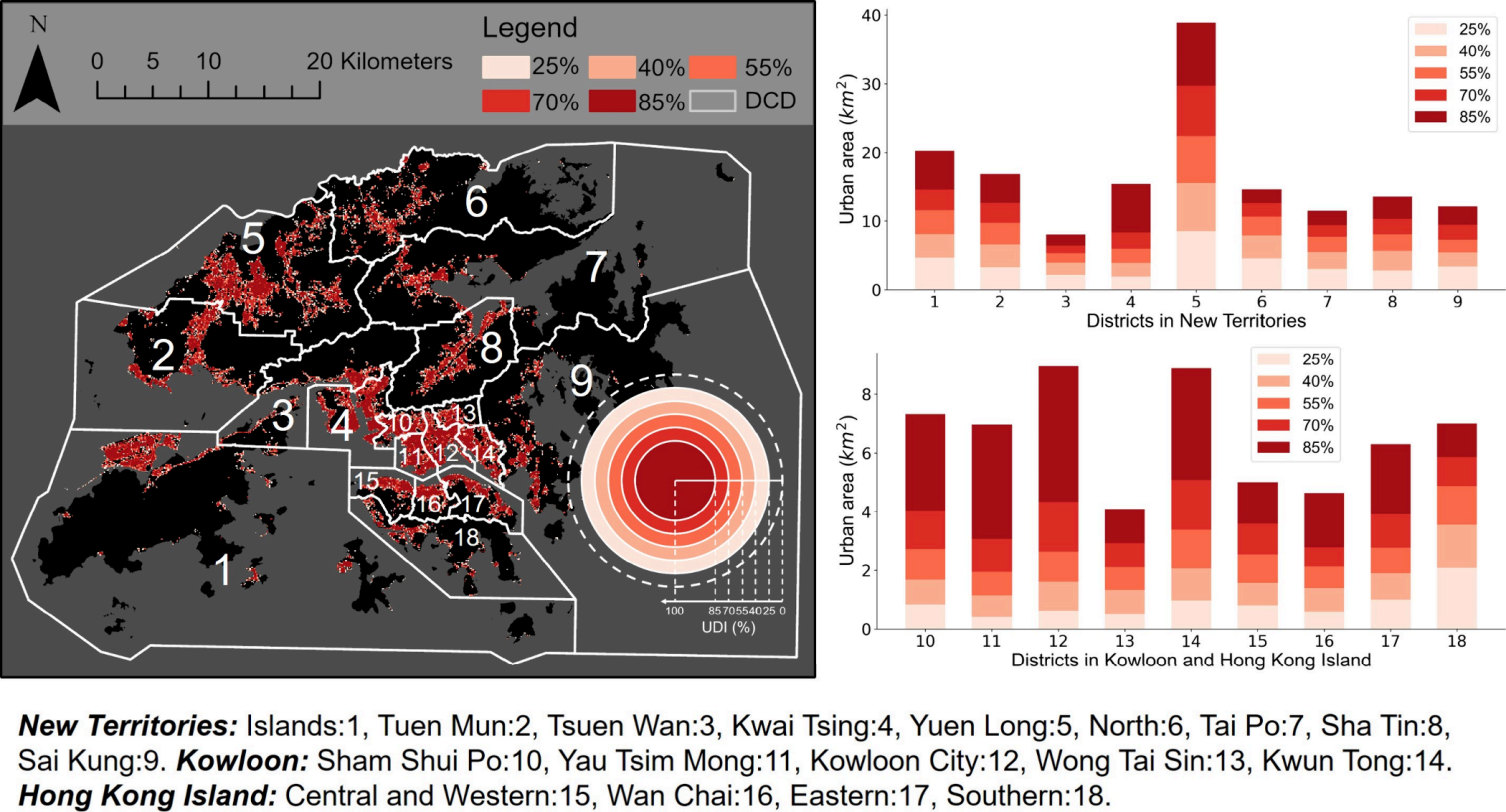
Distribution of urban areas in Hong Kong derived using the area dominance method and global impervious surface data. Publicly available boundary data was provided by the Hong Kong SAR Government.

### 2.2 Mobile Monitoring Particulate Matter (PM) Data

We conducted an extensive field survey to collect mobile air pollution data across four seasons in Hong Kong. A total of 800 participants from four districts, Central & Western, Kwun Tong, Kwai Tsing, and Sha Tin, were recruited to represent Hong Kong’s four traditional regions: Hong Kong Island, Kowloon, New Territories West, and New Territories East.^51^ Participants were equipped with low-cost portable devices (AirBeam) for two consecutive days, including one weekday and one nonweekday, between November 19, 2021, and April 6, 2023, with details documented in Supplementary Text S4. The study protocol was reviewed and approved by the Survey and Behavioral Research Ethics Committee of the Chinese University of Hong Kong (approval numbers: SBRE-19-123 and SBRE­(R)-21-005). Informed consent was obtained from all participants before data were collected from them. The AirBeam sensor can record PM_1_, PM_2.5_, and PM_10_ concentrations (unit of μg/m^3^) at one-second intervals and has been widely used in previous mobile environmental exposure studies.^52–54^ These sensors incorporate a built-in calibration algorithm, referencing the GRIMM EDM180, and we developed a random forest model to further calibrate the AirBeam sensor records, referencing the TSI DustTrak DRX Aerosol Monitor 8533,^55^ with details in Supplementary Text S5. To reduce variability, the original data were aggregated at 1 min intervals,^35^ resulting in a refined air pollution data set comprising approximately 1.894 million records, and 1.470 million records pertain to defined urban areas. We further interpolated these records using the empirical Bayesian kriging (EBK) method^56^ to produce an air pollution data set with a 10 m spatial resolution in urban areas across four seasons. In this study, the seasons are defined as follows: spring (December to February), summer (March to May), autumn (June to August), and winter (September to November). Finally, the seasonal average concentrations of PM_1_, PM_2.5_, and PM_10_ within 50 m buffers of streets in urban areas were used as air pollution measurements. The PM_2.5_ concentrations serve as the primary focus of this study, while the concentrations of PM_1_ and PM_10_ were incorporated primarily for sensitivity analyses. The results for the spatial distribution of PM_2.5_ concentrations across seasons are shown in Supplementary Text S6, Figure S2, and Table S1.

### 2.3 Historical Google Street View Images

We generated 197,012 sampling points along Hong Kong’s street network at 30-m intervals and retrieved corresponding street view images (SVIs) with timestamps and GPS coordinates through the Google Maps API. To avoid the geometric distortions of panoramic images, we adopted a smaller multiangle view^57^ (i.e., 90° field of view), and four images are needed at each sampling point to capture the full streetscape.^34,58–60^ In total, approximately 4.254 million monthly Google SVIs were collected, and about 3.232 million images in urban areas were used for further analysis, with a detailed statistic in Supplementary Text S7. Furthermore, these street-view images, collected from sampling sites with complete coverage across four seasons, were classified by season and matched to streets in Hong Kong to characterize street-level vegetation conditions in different seasons. To extract street greenspace, a cutting-edge deep learning semantic segmentation (i.e., Mask2Former algorithm) was employed, which has great performance on open data sets, such as ADE20K.^31^ We employed the Mask2Former algorithm trained on the ADE20K data set since it can identify vegetation types, including trees, plants, and grass. Combined with visual inspection, the tree type mainly corresponds to taller woody vegetation; the grass type primarily represents low and flat vegetation that continuously covers the ground surface; and the plant type generally refers to vegetation types with morphological characteristics between the two, such as shrubs, and we refer to the plant type as mid-level vegetation in this study. Based on the pixel-level segmentation of trees, mid-level vegetation, and grass, the Green View Index (GVI) was calculated as a metric for street greenspace,^29,61^ which ensures the unbiased assessment of the 360° environment around the sampling point, and the results were more robust than those obtained from a single direction measurement.^58^ The GVI is expressed as a percentage ranging from 0 to 1, with larger values indicating a greater density of visual greenspace, and is defined by the following equation:^34,62^

$$\mathrm{GVI}=\frac{\sum_{i=1}^{4}{\mathrm{Area}}_{g_{i}}}{\sum_{i=1}^{4}{\mathrm{Area}}_{t_{i}}}$$ 1

where the GVI represents the green view index at a sampling point; *i* indexes the four images captured at that point; Area_g_*i*__ is the number of green pixels (comprising trees, mid-level vegetation, and grass) in image *i*; and Area_t_*i*__ is the total number of pixels in image *i*. Finally, the average GVIs within 50-m buffers around streets were used as the greenspace measurements, with the unit of the proportion (%) of the image. The results are shown in Text S8, Figures S3–S6, and Table S1.

### 2.4 Covariates

Informed by prior literature, we incorporated a comprehensive set of covariates, including street structure, street length, building density, building height, altitude, slope, distance to coastline, population density, income, anthropogenic NMVOC emissions, background air pollution, temperature, rainfall, wind speed, relative humidity, and traffic volume.^2,59,62,63^ The street structure was quantified as the ratio of the average building height to the average width between buildings (H/W),^64^ calculated by intersecting the 50-m street buffer (Figure S7), with a dimensionless unit. Building height data were obtained from the 3D-GloBFP data set,^33^ while building shape data^65^ were sourced from the Lands Department. Street lengths were derived from the Road Network data^66^ provided by the Transport Department, with a unit of meters. Building density (dimensionless unit) and height (unit of meter) were quantified as the average area of building footprints and average height of buildings intersecting the 50-m street buffer, derived from building shape data and the 3D-GloBFP data set, respectively. These urban morphological factors were assumed to be time-invariant (i.e., their values were considered stable over the study period) as Hong Kong is a highly developed city, and street structure was given primary analytical focus. Altitude (unit of meter) and slope (unit of degree) were included as topographical confounders and were quantified based on the Slope analysis tool^67^ in QGIS and the Digital Terrain Model (DTM) data set^68^ provided by the Lands Department. Averaging their values intersecting the 50-m street buffer represents the street-level altitude and slope, respectively. The distance to the coastline (unit of meter) was quantified as the nearest distance from a street to the coastline using the Geological Map data set^69^ from the Civil Engineering and Development Department. Population density and income data, derived from the Census and Statistics Department,^70^ were also incorporated to account for their potential confounding effects on air pollution. The above-mentioned variables were considered time-invariant due to our short survey period. Anthropogenic NMVOC emissions were derived from the MIXv2 data set,^71^ which provides monthly NMVOC emission estimates of power, industry, residential, transportation, agriculture, and shipping sectors across Asia for 2010–2017, at a spatial resolution of 0.1° × 0.1° (unit of Mg/month/grid), and we used the most recent available monthly data from 2017 as representation. Background air pollution (i.e., PM_2.5_ and PM_10_, with units of μg/m^3^), temperature (unit of °C), rainfall (unit of mm), wind speed (unit of m/s), and relative humidity (dimensionless unit) data were collected from static monitoring stations in Hong Kong. These air pollution and meteorological data had high temporal resolution (daily records) and showed obvious seasonal variations, as shown in Supplementary Figure S8A–F. To address the limited spatial coverage of fixed monitoring stations, we used the advanced Empirical Bayesian Kriging (EBK) method to interpolate these point data into continuous 10 m-resolution raster surfaces for the urban areas of Hong Kong at the season level. Traffic volume data were derived from segmentation analyses of street view images, which identified vehicles such as cars, buses, trucks, and vans. They featured high spatial resolution (recorded every 30 m along the street network) and monthly temporal resolution. As shown in Supplementary Figure S8G, traffic volume also exhibited seasonal trends, with lower levels in summer and higher levels in other seasons. Anthropogenic NMVOC emissions, background air pollution, and street-level traffic volume were selected because they can account for baseline differences in air pollution at the regional and street levels, mainly driven by anthropogenic emissions, thereby enabling a more accurate identification of the net effect of urban greenspace on air pollution. Meteorological variables were incorporated to control for their spatial and season-related confounding effects. All covariate data were averaged at both the season and street levels to facilitate analyses.

### 2.5 Analytic Methods

Conventional linear models often struggle to accommodate high dimensionality and nonlinearity, particularly in large data sets, which can lead to biased and unstable effect estimates. To address these limitations, the double machine learning (DML) framework was proposed, grounded in the Frisch–Waugh–Lovell theorem, which integrates the strengths of machine learning algorithms with those of linear models.^72^ Consequently, the DML framework was applied in this study to estimate the unbiased average and heterogeneous effects of greenspace on air pollution. Specifically, DML is implemented in two stages, as illustrated in Figure 2. The model to be estimated is expressed as

Y=α+θ(W)T+g(X)+ϵ 2

where *Y* denotes the outcome variable (air pollutant); *T* represents the treatment variable (greenspace indicator); *X* indicates the covariates; *W* indicates the moderating factors; α and ϵ are the intercept and error term, respectively; θ­(*W*) denotes the average treatment effect (ATE) or conditional average treatment effect (CATE, heterogeneous effects) of *T*; and *g*(*X*) captures the confounding effects of *X* on *Y*. In the first stage, *Y* and *T* were predicted independently using machine learning algorithms to obtain their orthogonalized residuals, which effectively remove confounding effects. In the second stage, orthogonal residuals of *Y* and *T* were computed and regressed using conventional linear algorithms to derive debiased effect estimates of greenspace on air pollution. Through these stages, both unbiased average and heterogeneous effects (θ­(*W*)) were obtained.

**Figure 2:**
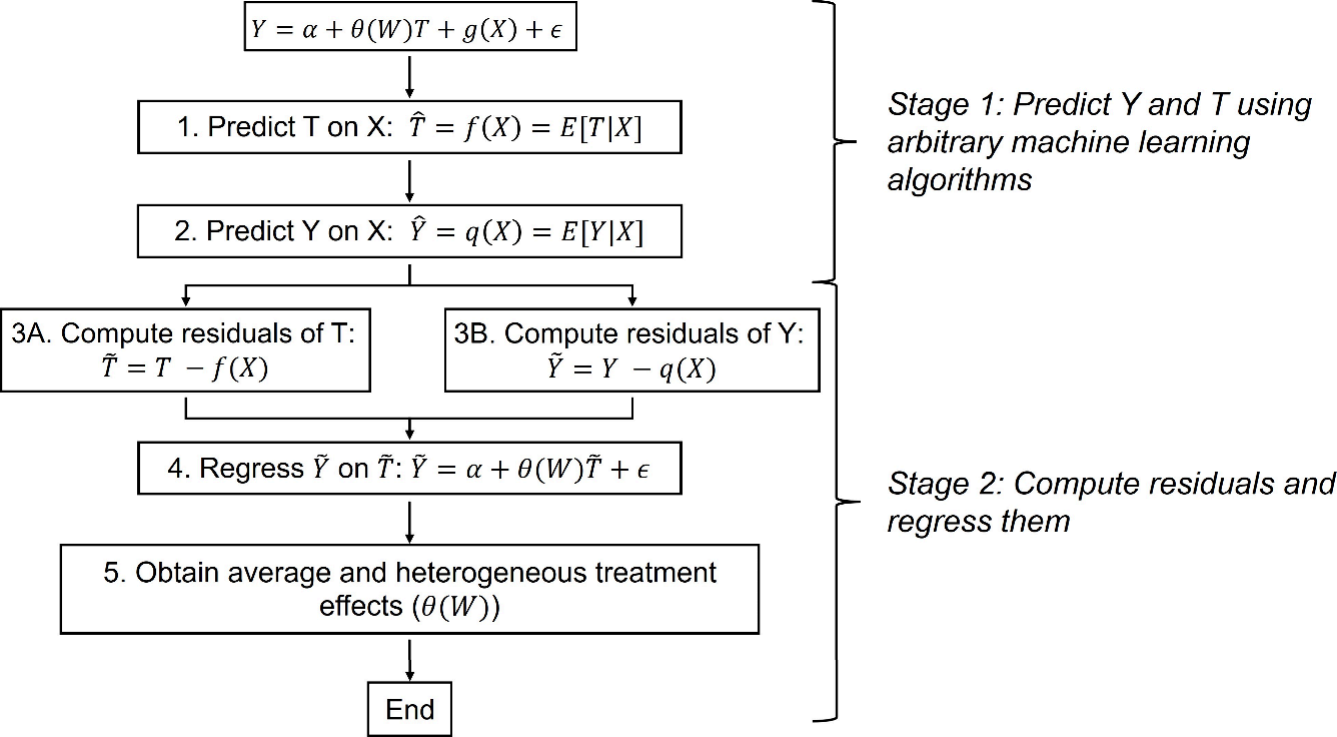
Flowchart of double machine learning based on the Frisch–Waugh–Lovell (FWL) theorem.

The first stage of the DML framework is critical, and only models capable of producing clean, orthogonalized residuals can support debiased estimates in the second stage. To ensure model robustness, we systematically compared seven model combinations: xgb-ols, lasso-ols, ridge-ols, dt-ols, rf-ols, gbdt-ols, and lgbm-ols. In the first stage, each model employed its corresponding algorithm: extreme gradient boosting (xgb), lasso regression (lasso), ridge regression (ridge), decision trees (dt), random forest (rf), gradient boosted decision trees (gbdt), and light gradient boosting machine (lgbm) model. The second stage uniformly applied an ordinary least-squares (ols) model with bootstrap techniques. The optimal machine learning model in the first stage was selected based on performance metrics, including two negative indicators (root-mean-square error (RMSE) and mean absolute error (MAE)) and one positive indicator (*R* ^2^). All models were trained and evaluated on seasonal and full-season data sets and applied defined measures (Supplementary Text S9) to avoid the overfitting problem. In the second stage, this study uniformly employed ordinary least-squares (OLS) regression to regress the orthogonalized residuals of air pollution and greenspace. Bootstrap resampling technique was used to construct confidence intervals (CIs) for the estimated effects, with a 90% confidence level selected in this study. Each OLS regression in the second stage was iterated 1,000 times. The outcome variable was PM_2.5_ concentration (μg/m^3^), and the treatment variables included the Green View Index (GVI) for overall greenspace, trees, mid-level vegetation, and grass. Predictors were the covariates defined in Section 2.4 and were preprocessed using a robust scaler (i.e., interquartile range (IQR) standardization) method.^73^ When estimating the effect of a specific vegetation type (e.g., tree), we included the other two vegetation types (e.g., mid-level vegetation and grass) as covariates in the first stage to control their potential confounding. While estimating the effect of overall greenspace (defined as the sum of tree, mid-level vegetation, and grass), we did not additionally control for these three vegetation variables because the treatment variable itself already represents total greenspace exposure. Additionally, when training models on the full-season data set, the seasonal factor was included as a predictor. Python was used for all training and evaluation processes. In the second stage, we also constructed the following equation to estimate the heterogeneous effect of greenspace on air pollution:

$$Y_{\mathrm{residual}}=\alpha +(\beta_{0}+\beta_{1}W)T_{\mathrm{residual}}+\epsilon$$ 3

here, *Y*_residual_ and *T*_residual_ represent the residuals of the outcome variable and treatment variable, respectively, obtained from the first stage of the DML framework. *W* is a moderating variable, which in this study indicates the street structure, characterized by the street height-to-width ratio and standardized using the robust scaler method. α and ϵ are the intercept and error term, respectively. β_1_ captures the moderating effect of *W*, and the term β_1_ *W* reflects the effect of greenspace on air pollution at different values of *W*, while β_0_ represents the effect when *W* takes its median value of 0.781. We applied the same approach used for estimating the ATE, that is, OLS combined with the bootstrapping technique to estimate heterogeneous effects. To further obtain effects of greenspace on air pollution and their CIs for each street (characterized by a unique *W* value), we conducted a simple slopes analysis. Specifically, by fixing the values of *W*, we utilized the estimated series of β_0_ and β_1_ values from eq 3 to calculate β_0_ + β_1_ *W*. This process was repeated for each street to derive street-level effects. Additionally, sensitivity analyses were conducted in terms of methods, indicators, and data sets and are detailed in Supplementary Text S10.

## 3 Results

### 3.1 Model Comparison

As described in Section 2.5, seven models were compared in the first stage of the DML framework: extreme gradient boosting (xgb), decision trees (dt), random forest (rf), gradient boosted decision trees (gbdt), light gradient boosting (lgbm), lasso regression (lasso), and ridge regression (ridge). Among these, lasso and ridge are conventional linear regression models, whereas the others are tree-based machine learning models. The optimal hyperparameters and performance metrics for these seven models are available on Zenodo. Based on these results, we visualized the performance of the seven models across the full season (Figure 3) and seasonal data sets (Supporting Information Figures S9–S12), using RMSE. All models demonstrated good generalization, and XGBoost generally outperformed the other six models in most cases. Using the result of the full-season data set as an example, the XGBoost model achieved mean *R* ^2^ values of 0.830, 0.771, 0.833, 0.670, and 0.636 in validation sets in predicting PM_2.5_, greenspace, trees, mid-level vegetation, and grass, respectively. While lasso and ridge regression showed nearly identical performance on both training and validation data sets, their overall goodness-of-fit was significantly lower than that of nonlinear machine learning models. Therefore, XGBoost was the most effective model in the first stage of the DML framework, which produced the cleanest and most orthogonal residuals for the greenspace indicators and PM_2.5_ concentrations, thereby providing a solid foundation for debiased effect estimates in the second stage. The contributions of different predictors (i.e., covariates) in predicting PM_2.5_ concentrations and greenspace indicators are available in Zenodo, and their results by using XGBoost models are visualized in Supporting Information Figures S13–S17 and described in Supporting Information Text S11.

**Figure 3:**
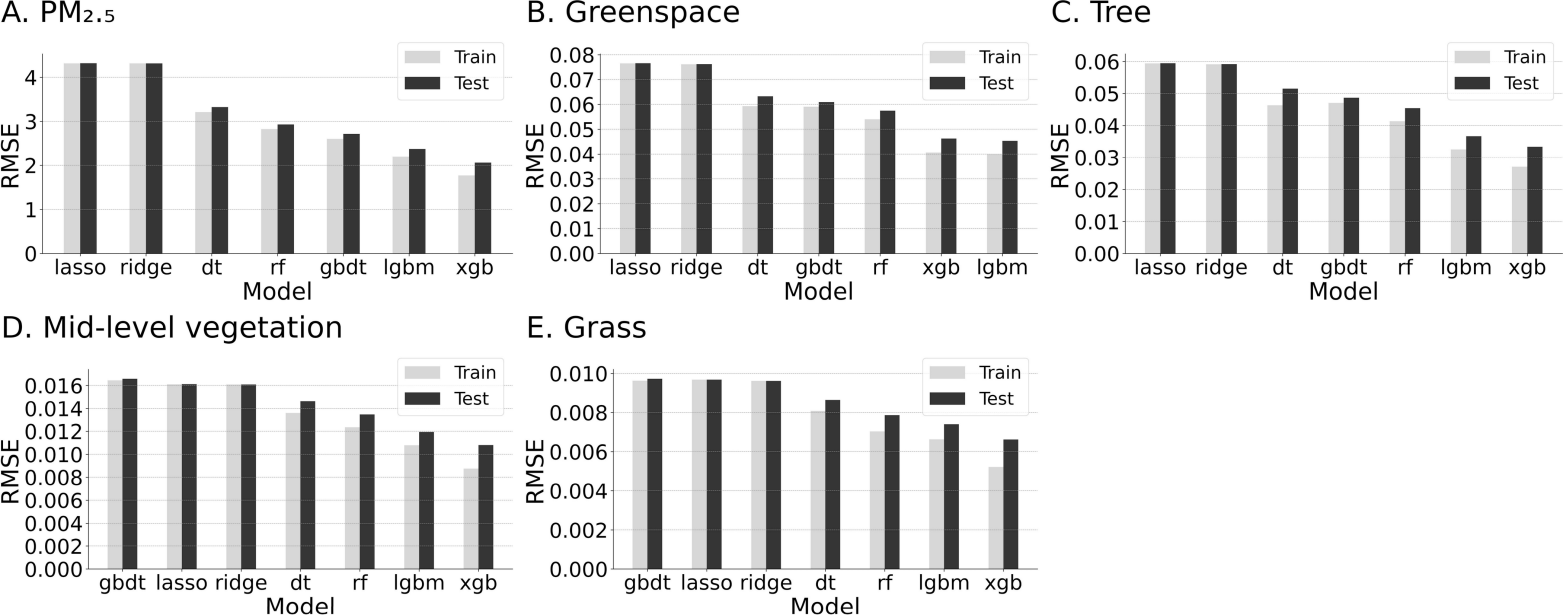
Model performance comparison for (A) PM_2.5_, (B) greenspace, (C) trees, (D) mid-level vegetation, and (E) grass in stage one of double machine learning using data in the full-season data set.

### 3.2 Influence of Seasons and Vegetation Types on Average Treatment Effects

The estimated effects of greenspace on air pollution using the conventional linear regressions and DML framework are presented in Supporting Information Table S2 and Figure 4. Regarding the ATEs estimated by the DML framework, we observed highly similar distributions of ATEs across different seasons and greenspace indicators when tree-based machine learning models were used in the first stage (Figure 4A,D,G,H,I), with slight differences in numerical ranges. Similarly, the DML framework involving linear models (Figure 4E,F) in the first stage produced nearly identical ATE distributions and numerical ranges to those generated entirely by traditional linear regressions (Figure 4B,C). However, significant differences emerged between the ATE distributions and ranges between linear models and machine learning models when they were used in the first stage of the DML framework. We attribute this discrepancy to the inability of traditional linear models to capture nonlinear relationships between outcome and prediction variables, resulting in biased ATE estimates. We further propose that the slight differences in ATE ranges arise from the varying model performance of the machine learning algorithms used in the first stage. Consequently, this study adopted the xgb-ols model combination, that is, employing the XGBoost model in the first stage and the OLS regression in the second stage. A negative ATE indicates that greenspace mitigates PM_2.5_ levels, whereas a positive ATE suggests an exacerbating effect. The effect size quantifies the change in PM_2.5_ concentrations per unit change in the proportion of greenspace in street-view images. Overall, we found that the effect of street greenspace reflects the combined effects of different vegetation types. Specifically, regarding street greenspace, it exhibited a significant negative ATE (−0.641, 90% CI: −0.913 to −0.372) on PM_2.5_ levels, while seasonally, street greenspace only exhibited significant negative ATEs on PM_2.5_ levels in spring (−1.219, 90% CI: −1.672 to −0.757) and autumn (−0.751, 90% CI: −1.260 to −0.226). In the summer, street greenspace significantly exacerbated PM_2.5_ pollution, with a mean ATE of 0.390 (90% CI, 0.051–0.713). Regarding street trees, it only exhibited a significant positive ATE (0.762, 90% CI: 0.347–1.178) on PM_2.5_ levels in summer, while it was not significant in spring, autumn, winter, and full-season data sets. Regarding street mid-level vegetation, it exhibited a significant positive ATE (3.480, 90% CI: 0.457–6.596) on PM_2.5_ levels in winter. In spring, street mid-level vegetation significantly mitigated PM_2.5_ pollution, with a mean ATE of −2.275 (90% CI, −4.132 to −0.298). Regarding street grass, it exhibited significantly negative ATEs in the full-season data set (−5.890, 90% CI: −7.893 to −3.940), spring (−8.464, 90% CI: −11.142 to −5.911), and autumn (−6.506, 90% CI: −10.098 to −3.075), while it was not significant in summer and winter. Previous studies have shown that the effects of vegetation on air pollution are multifaceted. On the one hand, vegetation can reduce particulate matter through deposition; on the other hand, it may also exacerbate particulate pollution through the emission of BVOCs and the generation of adverse aerodynamic effects.^6,7^ We therefore hypothesize that the heterogeneous effects of different vegetation types across seasons essentially reflect the trade-offs among the three mechanisms.

**Figure 4:**
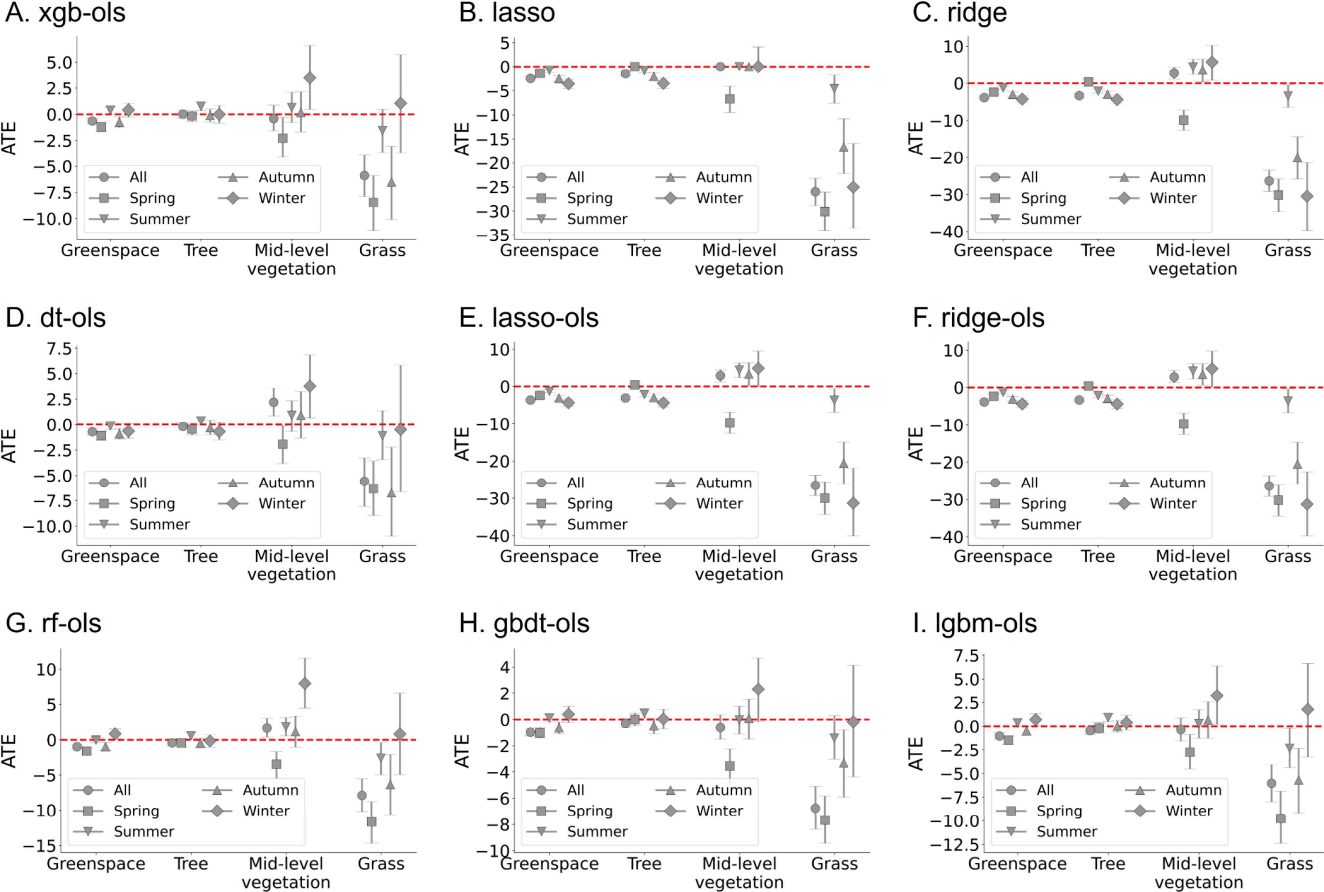
Comparison of model combinations: (A) xgb-ols, (B) lasso, (C) ridge, (D) dt-ols, (E) lasso-ols, (F) ridge-ols, (G) rf-ols, (H) gbdt-ols, (I) lgbm-ols, for estimating ATE of overall greenspace, trees, mid-level vegetation, and grass on PM_2.5_ concentrations. Different markers in the figure indicate ATE estimates derived from seasonal and full-season data sets.

### 3.3 Moderating Effects of Street Structures on Heterogeneous Effects

Following Section 3.2, we conducted an analysis of the heterogeneous effects of greenspace on PM_2.5_ pollution, moderated by street structure (H/W: the street height-to-width ratio) using the xgb-ols DML framework. The average estimates and 90% CIs for the parameters β_0_ and β_1_ in Equation 3 are summarized in Supplementary Table S3, and the results of the simple slopes analysis are visualized in Figure 5, and the significant and nonsignificant partitioned intervals of estimated effects are recorded in Supplementary Table S4. From these results, we observed that, for overall greenspace, street structure (H/W) exhibited significant negative moderating effects in full-season, summer, and autumn, while showing significant and positive moderating effects in spring. For street trees, the results indicated that in summer, street structure (H/W) exhibited a significant positive moderating effect. While in autumn, street structure (H/W) exhibited a significant negative moderating effect. For mid-level vegetation, the results indicated that in spring, street structure (H/W) had a significant negative moderating effect. While in winter, street structure (H/W) showed a significant positive moderating effect. For street grass, street structure (H/W) exhibited a significant positive moderating effect in both full-season and autumn. While in spring, street structure (H/W) exhibited a significant negative moderating effect. It should be noted that this study did not consider heterogeneity effects with a shorter significance interval. These findings suggest that the effects of different vegetation types across seasons may vary substantially across street canyons with different height-to-width ratios. A potential explanation is that street height-to-width ratio alters ventilation conditions and pollutant dispersion processes within the canyon, thereby shaping the net effects of different vegetation types on air pollution across seasons.^6,74^ Ultimately, these net effects are likely determined by the relative magnitudes among vegetation-mediated deposition, aerodynamic effects, and BVOC emissions within street canyons. This result has important implications for avoiding one-size-fits-all greening strategies and for advancing more targeted urban greening practices.

**Figure 5:**
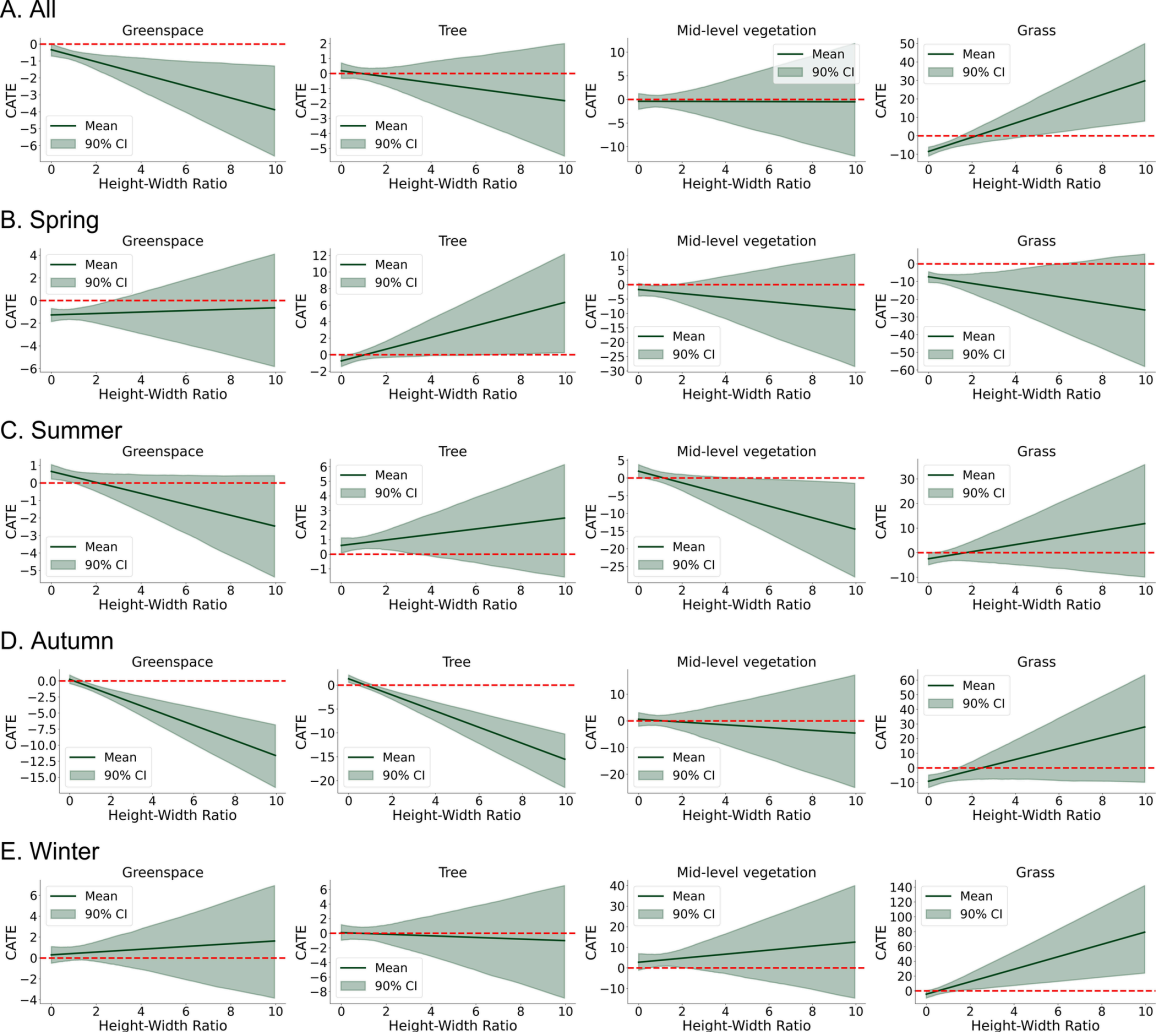
Heterogeneous effects of greenspace on air pollution, moderated by the street height-to-width (H/W) ratio, across seasons: (A) all, (B) spring, (C) summer, (D) autumn, (E) winter.

### 3.4 Sensitivity Analyses

In addition to the sensitivity analysis of method selection presented in Section 3.2, we also conducted sensitivity analyses using the xgb-ols DML framework based on indicator and data set selection. The results of hyperparameters and performance for XGBoost in the first stage and estimated effects in the second stage are available in Zenodo. Regarding the sensitivity analysis of indicator selection, first, we compared three air pollutants (i.e., PM_2.5_, PM_1_, and PM_10_), as shown in Supporting Information Figure S18. We found a consistent trend in the full-season data set and winter: both overall greenspace, trees, and mid-level vegetation exhibited stronger mitigation effects on PM_1_ than on PM_2.5_ and stronger effects on PM_2.5_ than on PM_10_. In other seasons, the effects of greenspace indicators on different air pollutants were inconsistent, which may be attributed to the reduced statistical power of smaller sample sizes or to significant seasonal variations. Second, we compared three types of greenspace representation, two 3D metrics, overall greenspace from street view images (SVI-derived greenspace) and canopy height of vegetation derived from satellite images, and one 2D metric, NDVI, as presented in Supporting Information Figure S19. We found that only NDVI consistently and significantly mitigated air pollution (full season, autumn, and winter), while SVI-derived greenspace and canopy height demonstrated more complex results. Regarding the sensitivity analysis of data set selection, we compared two data sets characterizing traffic volume: the proportion of vehicles derived from street view images (SVI-derived traffic) and the number of vehicles recorded by street detectors (detector-derived traffic). Due to the limited streets matched with the two data sets, we further compared results from these matched streets with all streets using SVI-derived traffic, as shown in Supporting Information Figure S20. On the one hand, we observed a high degree of consistency in the estimated effects of greenspace on air pollution across seasons when using SVI-derived traffic and detector-derived traffic for the limited matched streets. On the other hand, significant differences emerged when comparing these findings to results based on SVI-derived traffic across all of the streets. This discrepancy is likely due to substantial heterogeneity among the streets. Therefore, in the core analysis of this study, we used the SVI-derived traffic in all streets as the control variable for traffic volume.

## 4 Discussion

The heterogeneous effect of urban greenspace on air pollution is multifaceted and influenced by seasons, vegetation types, and urban morphology. Previous studies have not thoroughly addressed this complexity because of limitations in data, methods, and ecological validity. To bridge this gap, this study integrated mobile monitoring air pollution data, historical street view imagery data, and 3D urban building data, employing a DML framework to disentangle these heterogeneous effects and investigate influences from seasons, vegetation types, and street structures.

### 4.1 Explaining the Average Treatment Effects of Urban Greenspace

According to Section 3.2, we found that the ATEs of urban greenspace on air pollution were not constant. On the contrary, both their magnitudes and directions were jointly influenced by the vegetation type and season. This finding indicates that focusing solely on the capacity of urban greenspace to mitigate air pollution (e.g., deposition) is insufficient.^4,63^ Instead, the net effect of greenspace reflects the balance among multiple physicochemical processes, including BVOC emissions, aerodynamic effects, and pollutant deposition. Specifically, vegetation-derived BVOCs, such as isoprene and monoterpenes, can enhance the atmospheric oxidative capacity and promote secondary organic aerosol (SOA) formation, thereby exacerbating particulate pollution.^8,58^ Our supplementary analyses (Text S12 and Table S5) further support this interpretation, showing that higher BVOC emissions weakened the net pollution mitigation effect of vegetation. In addition, vegetation-induced aerodynamic effects, including reduced wind speed and weakened vertical turbulent exchange, can suppress pollutant dispersion and lead to pollutant accumulation within street canyons; by contrast, vegetation can also reduce air pollution through deposition, for example, by intercepting particulate matter on leaf surfaces.^6,75,76^

By vegetation type, using the full-season data set as an example, we found that the capacity to mitigate air pollution declined from grass to mid-level vegetation to trees, with some vegetation types even exacerbating pollution. This variation may be related to differences in the three-dimensional structure and associated aerodynamic effects. Trees and mid-level vegetation have more pronounced vertical structure, which can alter airflow patterns within street canyons and suppress ventilation, thereby hindering pollutant dispersion. A review reported that, relative to treeless street canyons, street canyons with trees typically exhibit lower wind speeds and higher pollutant concentrations.^6^ For mid-level vegetation, such as hedges, although it can also reduce wind speed within street canyons, the local vortices it induces may, in some cases, offset part of these adverse effects and thereby improve air quality.^6^ However, other studies have shown that hedges can also exacerbate air quality due to their permeability and airflow alteration, and this adverse effect becomes more pronounced as hedge height increases.^77^ On the contrary, because grass is low in height and relatively flat in structure, it interferes less with street canyon ventilation and is therefore more likely to reduce air pollution through processes such as deposition.^9,78^ In addition, compared with grass, trees and mid-level vegetation, such as shrubs, generally have higher BVOC emission potentials^37,79^ and are more likely to promote secondary organic aerosol formation, thereby exacerbating air pollution.^8,58^

Urban greenspaces also exhibited pronounced seasonal variation in their effects on air pollution. Using the overall greenspace as an example, we found that it generally exacerbated air pollution in the summer and winter but mitigated it in the spring and autumn. This seasonal variation may reflect shifts in the relative magnitudes of BVOC emissions, pollutant deposition, and the aerodynamic effects. Specifically, during the summer, urban vegetation typically exhibits stronger BVOC emissions, which may promote the formation of secondary pollutants, thereby exacerbating air pollution.^44,45^ This interpretation is further supported by BVOC level data from the monitoring stations (Supporting Information Figures S21 and S22). In the winter, BVOC emissions from vegetation are lower, and phenological changes weaken the capacity of vegetation to remove pollutants through deposition. Under these conditions, its aerodynamic effects, such as obstructing airflow within street canyons, may become more prominent. At the same time, lower winter wind speeds (Figure S23) may further amplify the adverse aerodynamic effects of vegetation on air quality.^39,80^ Conversely, in spring and autumn, vegetation BVOC emissions remain at moderate levels (Figure S22), while urban ventilation conditions are relatively favorable (Figure S23). Under such conditions, the pollutant removal effect of vegetation through deposition may dominate, thereby leading to a net mitigation of the air pollution.

### 4.2 Explaining the Heterogeneous Effects of Urban Greenspace

According to Section 3.3, we further found that street structure, as characterized by the street height-to-width (H/W) ratio, significantly moderated the heterogeneous effects of urban greenspace on air pollution. This heterogeneity may likewise arise from the combined influence of BVOC emissions, pollutant deposition, and the aerodynamic effects. Overall, variation in the street height-to-width ratio primarily alters ventilation and pollutant dispersion within street canyons,^6,74^ thereby shaping the net effects of different vegetation types on air pollution across the seasons.

For trees, the pollution-exacerbating effect in summer intensified with an increasing street height-to-width ratio. One possible explanation is that BVOC emissions from trees are stronger in the summer, making BVOC-driven pollution formation more pronounced.^44,45^ At the same time, a higher street height-to-width ratio can suppress ventilation and weaken air exchange and pollutant dispersion within the canyon,^6,74^ thereby facilitating the accumulation of BVOCs and further amplifying the adverse effect of trees on air pollution. In contrast, in autumn, the pollution-mitigating effect of trees became stronger as the street height-to-width ratio increased. A possible explanation is that, under the moderate BVOC emissions and relatively favorable ventilation conditions in Hong Kong in autumn, the removal of pollutants by trees through deposition is more likely to dominate. Against this background, although a higher street height-to-width ratio reduces ventilation efficiency, it may also prolong the pollutant residence time within the canyon,^8,12^ thereby increasing the opportunity for contact with tree surfaces and subsequent removal. In spring, winter, and full season, the effects of tree BVOC emissions, deposition, and aerodynamics may offset one another, and accordingly, neither its ATE nor heterogeneous effects were statistically significant.

For mid-level vegetation, the pollution-mitigating effect in spring became stronger as the street height-to-width ratio increased, suggesting that its net effect may be dominated by deposition. A larger street height-to-width ratio can reduce ventilation efficiency and prolong pollutant residence time,^8,12^ thereby making the net removal effect of mid-level vegetation more pronounced. By contrast, in winter, the pollution-exacerbating effect of mid-level vegetation intensified with increasing street height-to-width ratio, implying that its net effect may be governed by aerodynamic effects. Under these conditions, the obstruction of airflow by mid-level vegetation becomes more pronounced in street canyons with high height-to-width ratios, thereby hindering pollutant dispersion^6,74^ and potentially amplifying its adverse effect on air quality. In summer, autumn, and the full season, the effects of BVOC emissions, deposition, and aerodynamics may offset one another, and accordingly, both the ATE and heterogeneous effects of mid-level vegetation remained relatively limited.

For grass, the pollution-mitigating effect in the spring intensified with an increasing street height-to-width ratio, suggesting that the net effect of vigorously growing grass may be dominated by deposition. A higher street height-to-width ratio is typically associated with weaker ventilation and longer pollutant residence time, thereby favoring the expression of the pollutant removal effect of grass through deposition.^9^ Conversely, in autumn and full season, the pollution-exacerbating effect of grass became stronger as the street height-to-width ratio increased. In light of the existing literature, this pattern more likely reflects the following mechanism: although the effects of grass on air quality are mainly through deposition, its depositional capacity is constrained by phenological variation and may weaken in the autumn. At the same time, as the street height-to-width ratio increases, ventilation exchange is reduced and pollutants accumulate more readily, such that the net depositional benefit of grass is more easily offset, resulting in a weakened net improvement effect or even a relatively adverse effect.^8,76^ In summer and winter, the BVOC-related, depositional, and aerodynamic effects of grass may counterbalance one another, and accordingly, neither its ATE nor heterogeneous effects were statistically significant.

### 4.3 Explaining Double Machine Learning Framework and Sensitivity Analyses

In Sections 3.1 and 3.2, we systematically compared the results of different model combinations in the DML framework and with those of traditional linear models. Our findings suggest that the DML framework demonstrates robustness and superiority. First, the distributions of the effects of greenspace on air pollution, using the DML framework, are remarkably similar, with only minor numerical differences. When we replaced the first-stage machine-learning models with linear models, the estimated effects closely matched those obtained using traditional linear regression. These consistencies underscore the robustness of the DML framework. Second, significant differences emerged between the estimated effects derived from the DML framework (with machine-learning algorithms in the first stage) and those from traditional linear models. Notably, traditional linear models tend to yield large estimates. However, theoretically, these results are likely to be biased and misleading. Otherwise, the estimates from the two approaches would exhibit a high similarity. We argue that traditional linear models fail to capture nonlinear relationships among variables, while the DML framework leverages the power of machine learning to effectively eliminate confounding influences, yielding unbiased and valid estimates. These findings highlight the superiority of the DML framework. In Section 3.4, we conducted sensitivity analyses on indicator and data set selection within the DML framework. First, we found that the effects of urban greenspace on air pollution vary across different pollutants, underscoring the need for pollutant-specific analyses. Second, we observed that different types of greenspace indicators reveal distinct relationships, which highlight the limitations of traditional two-dimensional greenspace indicators (NDVI). Third, we examined traffic volume as a confounding factor using two data sets (i.e., street-view imagery and street traffic detectors) and found high similarity in the estimated effects. This result underscores the validity of street-view imagery in characterizing the urban street traffic volume.

### 4.4 Limitations

This study acknowledges several limitations that merit further exploration. We acknowledge that this study was unable to directly include BVOC emissions as a control variable in the main analysis or to rigorously quantify their contribution because reliable, publicly available BVOC emission data at the street scale are currently unavailable. Furthermore, the mechanism explanation of our results is mainly based on a comprehensive inference of the trade-offs among vegetation-mediated BVOC emissions, pollutant deposition, and aerodynamic effects. We also acknowledge that the classification of vegetation type based on street-view images does not represent a strict botanical taxonomy but rather a visually defined semantic classification based on the ADE20K’s annotation scheme, and seasonal greenspace measurements derived from street-view images can serve only as proxy measures of phenological variation as well, rather than strict biological phenology. Our measurement of control variables for background air pollution and meteorological factors relied on a limited number of fixed monitoring stations supplemented by interpolation methods, which may compromise the accuracy of estimated effects associated with street greenspace. The estimated effects based on the double machine learning framework are debiased effects under certain assumptions, while not strict causality, which required integrated longitudinal observations into the DML framework. This investigation focused solely on the debiased relationship between street greenspace and air pollution, without linking these factors to human health behaviors (e.g., physical activity) and outcomes (e.g., psychological well-being).

## Supplementary Material



## Data Availability

The historical Google Street View images were provided by Google under license. The authors do not have permission to provide these images to others under the license agreement. The mobile air pollution monitoring data is not publicly available due to participant privacy issues. The three-dimensional building data are openly available in Building Height of Asia in 3D-GloBFP at https://zenodo.org/records/12674244. The global 10 m resolution impervious surface data is openly available in Mapping 10-m global impervious surface area (GISA-10m) using multi-source geospatial data at https://zenodo.org/records/5791855. Street length, slope, altitude, background air pollution, temperature, rainfall, wind speed, relative humidity, population density, and income are openly available in Open Data of Hong Kong at https://data.gov.hk/en/. The results of parameters, performance, and feature importance for the applied model in the main text and average treatment effects estimated for sensitivity analyses are stored in a Zenodo dataset: https://doi.org/10.5281/zenodo.19148381.

## Notes

Auxiliary texts for related content of urban area extraction, distributions of air pollution and greenspace, and detailed descriptions of data set, sensitivity analysis, measures avoiding overfitting problem, and BVOC-related analyses; auxiliary figures for the study area, schematic of street structure calculation, spatial distributions of air pollution and greenspace, temporal distributions of covariates, model performance, feature importance, sensitivity, and BVOC-related analyses; and auxiliary tables for descriptive results of air pollution and greenspace, average treatment effects, heterogeneous effects, and BVOC-related analyses (PDF)
