# Supplementary material for: Reassessing the Heterogeneous Effects of Greenspace on Air Pollution Conditioned on Seasons, Vegetation Types, and Street Structures
Source: Environ Sci Technol. 2026 May 27;60(22):15888–900. doi: 10.1021/acs.est.5c15520 (PMC13262047; doi:10.1021/acs.est.5c15520)
Supplement: Supplementary file 1 [file es5c15520_si_002.pdf]

Supporting Information for

Reassessing the Heterogeneous Effects of Greenspace on Air Pollution Conditioned on Seasons, Vegetation Types, and Street Structures

Zhenchuan Yang [a], *Mei-Po Kwan* [a, b*], *Yan Zhang* [a]

[a] Institute of Space and Earth Information Science, Fok Ying Tung Remote Sensing Science Building, The Chinese University of Hong Kong, Shatin, Hong Kong, China.

[b] Department of Geography and Resource Management, The Chinese University of Hong Kong, Shatin, Hong Kong, China.

* Mei-Po Kwan (Corresponding Author)

Email: mpk654@gmail.com

This file includes:

Texts S1-S12

Figures S1-S23

Tables S1-S5

Supporting Information Texts

Text S1. The threefold rationales for refining the study area to the urban areas of Hong Kong.

We refined the study area to urban areas of Hong Kong, reflecting considerations of research design and data availability. First, we aim to examine the moderating role of street structure on the greenspace-air pollution relationship, while rural areas with a sparse street network are unsuitable. Second, as street view imagery (SVI) is captured by vehicles driving on streets, rural areas with limited streets lack the comprehensive SVI coverage. Finally, our air pollution data were acquired from local participants wearing portable devices, whose daily mobility mainly covered the urban areas of Hong Kong. By strategically focusing on the urban areas, we effectively minimize the potential biases arising from the above-mentioned constraints, thereby ensuring a more representative dataset.

Text S2. Extraction details for urban areas using the Urban Development Intensity (UDI) Indicator and the area dominance method.

In this study, the UDI indicator is computed as follows:

$$UDI_i = \frac{S_{Impervious\ surface,i}}{S_i} \times (S1)$$

where i indexes the image elements of a 100m resolution raster map of Hong Kong; UDI_i denotes the UDI value for the ith element; $S_{Impervious\ surface,i}$ represents the area of impervious surfaces within the ith element, derived from 10m resolution data; and S_i denotes the total area of the ith element. Urban areas are identified using an area dominance method, with image elements for which $UDI_i > 25\%$ classified as urban, and those at or below this threshold classified as rural. Urban areas were further stratified into five levels using a UDI interval of 15%: level 1 ($UDI_i = 85 - 100\%$), level 2 ($UDI_i = 70 - 85\%$), level 3 ($UDI_i = 55 - 70\%$), level 4 ($UDI_i = 40 - 55\%$), and level 5 ($UDI_i = 25 - 40\%$).

Text S3. Interpretation of the distribution of extracted urban areas in Hong Kong.

Using the area dominance method and global impervious surface data, we delineated Hong Kong's urban areas and categorized them into five urban development intensity (UDI) levels, as illustrated in Figure 1 in the main text. In the New Territories, the Yuen Long district (5) exhibits the largest urban area, primarily because it is the third largest district in Hong Kong and features expansive plains conducive to urban development. In this district, the government has planned two new towns, i.e., Yuen Long and Tin Shui Wai, along with the Hung Shui Kiu/Ha Tsuen New Development Area. In the Kowloon region, Kowloon City (12) and Kwun Tong (14) contain the largest urban areas, reflecting their status as the two largest districts within the more developed region. On Hong Kong Island, the Southern district (18) possesses the greatest urban area, attributable to its size being equivalent to the combined area of the other three districts (i.e., Central and Western (15), Wan Chai (16), and Eastern (17)). Moreover, an analysis of the proportions of different UDI levels reveals that the Southern district (18) exhibits lower proportions of high UDI areas compared with the other three districts. Similarly, the New Territories demonstrates lower proportions of high UDI levels, suggesting that its urban development lags behind that of Kowloon and Hong Kong Island, despite its larger urban area. This pattern aligns with Hong Kong's historical development, wherein the areas flanking Victoria Harbor, the five districts in Kowloon and the three northern districts on Hong Kong Island served as the traditional core, thereby achieving the highest levels of urban development.

Text S4. The details of how mobile monitoring data on air pollution were collected from the participants in our study.

Participants were equipped with low-cost portable devices (AirBeam) for two consecutive days, including one weekday and one non-weekday, between November 19, 2021, and April 6, 2023. The recruited participants were distributed throughout the study period, and on average, we conducted one round of experiments each week during the study period, involving approximately 20 participants per round. The data collection process was as follows: (1) On the day before the experiment, all participants attended a briefing session. This session introduced the study's objectives, experimental procedures, and the operation of the air pollution monitoring devices (AirBeam); (2) The air pollution monitoring experiment lasted continuously for 48 hours, with the start day scheduled on a Friday or Sunday to ensure one weekday and one non-working day were included; (3) Participants were instructed not to alter their daily routines and to wear the air pollution monitors continuously during the experiment, allowing us to capture their exposure to air pollution in urban environments; (4) Throughout the experiment, our research team remotely monitored the air pollution levels recorded by each mobile device. This allowed us to address sudden or potential issues, such as devices shutting down due to low battery; (5) After 48 hours, the air pollution monitoring experiment concluded, and all devices were collected.

Text S5. The description of the calibration of air pollution data recorded by Airbeam sensors.

We utilized AirBeam sensors, which are widely employed in mobile environment exposure studies[1–3]. These sensors incorporate a built-in calibration algorithm, referencing the GRIMM EDM180[4]. The air pollution data we obtained were already calibrated through this algorithm. Previous studies have demonstrated the reliability of AirBeam sensors for measuring particulate matter (PM). For example, Michael and Lim compared AirBeam readings with those from the TSI DustTrak DRX Aerosol Monitor 8533 in a concentrated air pollutants chamber. Their results revealed a highly linear relationship ($R^2 = 0.89$ for $PM_{2.5}$ and $R^2 = 0.88$ for PM_1)[4,5]. To further ensure measurement reliability across different urban micro-environments, we conducted field investigations in Hong Kong (our study area), including office (indoor), office (outdoor), MTR station (platform), MTR station (lobby), and seaside locations. We used a TSI DustTrak DRX Aerosol Monitor 8533 as the reference instrument and tested five AirBeam2 sensors. The results demonstrated high consistency in 1-min PM concentration measurements across the AirBeam2 sensors in all urban micro-environments, with correlation coefficients ranging from 0.73 to 0.96. However, we observed a lower linear relationship between AirBeam and DustTrak sensors compared to the prior study[5], potentially due to the AirBeam2's sensitivity to high-humidity environments[6–9]. To mitigate this problem, we developed a machine learning algorithm (random forest) to calibrate the AirBeam measurements. The calibrated PM concentrations showed a strong alignment with DustTrak measurements, achieving R^2 values ranging from 0.90 to 0.95[4].

Text S6. Differences in air pollution across seasons in urban areas of Hong Kong.

Using mobile air pollution monitoring data combined with the empirical Bayesian kriging (EBK) method, we simulated the spatial distributions of air pollutants across four seasons in urban areas of Hong Kong (Figure S2 and Table S1). Our results indicate that air pollution exhibits clear seasonal variations, with lower $PM_{2.5}$ concentrations in summer (mean = 9.110 μg/m^3) compared to spring (mean = 10.729 μg/m^3), autumn (mean = 14.711 μg/m^3), and winter (mean = 13.439 μg/m^3). These findings are consistent with data from Hong Kong's 18 static monitoring stations (Table S1). Moreover, seasonal spatial patterns reveal that in summer (Figure S2B), the districts of Sai Kung (9) and Kwun Tong (14) experience the most severe air pollution. In spring (Figure S2A), Central and Western (15) and Wan Chai (16) are the most affected, while in both autumn (Figure S2C) and winter (Figure S2D), Sha Tin (8) is identified as the most polluted district. These patterns may be attributed to intensive commercial and industrial activities, as well as high levels of human and vehicular movement in these areas.

Text S7. The descriptive statistics of seasonal street view images obtained in this study.

Initially, we attempted to retrieve street view images for all sampling points during the study period, with a temporal resolution of one month. However, due to the relatively short study period, there were significant gaps in the availability of street view images. Considering that Hong Kong is a highly developed city with a stable urban landscape over time, we extended the search period back to 2008 to obtain almost all available Google street view images within Hong Kong. As a result, out of a total of 197012 sampling points, 90439 (45.905%) were found to have street view data in the urban areas of Hong Kong. Specifically, among 90439 sampling points, 2780 (3.074%) sampling points had images for only one season; 7020 (7.762%) sampling points had images for two seasons; 26548 (29.355%) sampling points had images for three seasons; and 54091 (59.809%) sampling points had images for all four seasons. Furthermore, the street view imagery data from these sampling points will be averaged within the 50-meter buffer zone of the street, serving as a representation of the relevant indicators of this street based on the street view imagery data. Notably, we found that a total of 18557 streets had street view images available for all four seasons.

Text S8. Differences in street greenspace across seasons and vegetation types.

Using historical street view images and the Mask2Former algorithm, we calculated the greenspace level for each street in the urban areas of Hong Kong (Table S1). Our analysis reveals that street greenspace exhibits seasonal variability, with higher mean values in summer (0.132) and autumn (0.130) compared to spring (0.119) and winter (0.117). Moreover, Figure S3 shows that the spatial distribution of greenspace is consistent across seasons, with the North (6), Tai Po (7), and Sai Kung (9) districts consistently exhibiting the highest levels. In addition, we computed and mapped the seasonal distributions of various vegetation types, including trees, mid-level vegetation, and grass, across streets (Figures S4, S5, and S6, respectively), with the descriptive statistics provided in Table S1. Notably, street trees and mid-level vegetation have higher values in summer and autumn than in spring and winter, while street grass is more prevalent in spring, summer, and autumn than in winter. The districts of Islands (1), Yuen Long (5), North (6), Tai Po (7), Sha Tin (8), Sai Kung (9), and Southern (18) consistently rank among the top three for street trees, mid-level vegetation, and grass across different seasons. Overall, districts exhibiting higher levels of greenspace, characterized by abundant trees, mid-level vegetation, and grass, are primarily concentrated on the outer edges of the New Territories and in the southern part of Hong Kong Island. These spatial patterns may be attributable to lower urban development, favorable ecological endowments, and effective greening policies and practices.

Text S9. The measures to resolve overfitting problems in the first stage of the DML framework and consequently ensure the robustness of estimates in the second stage.

Overfitting remains a concern in machine learning and can compromise model selection based solely on these metrics. To address this, we implemented 5-fold cross-validation during training and evaluation and further fine-tuned hyperparameters to prevent overfitting. Specifically, we ensured that: (1) The ratio of the average performance on the validation set to the training set (in terms of RMSE or MAE) did not exceed 1.5; (2) The absolute difference of the average R^2 values between the validation and training sets did not exceed 0.2. If these conditions were met, the model was deemed not to be overfit in this study. We first applied the Bayesian optimization-based tool Optuna[10] to search for optimal hyperparameters within predefined hyperparameter spaces, aiming to achieve superior model performance. Using the best hyperparameter combinations identified, we conducted five-fold cross-validation[11], randomly splitting the dataset into five subsets. For each iteration, four subsets (80%) were used for training, and the remaining subset (20%) was used for validation, with the process repeated five times. After training, we evaluated all models for overfitting. In cases of overfitting, we further refined the hyperparameter search space and repeated the training and validation process under stricter conditions.

Text S10. Detailed description of the sensitivity analysis in this study.

To ensure the robustness of the study's results, we conducted a systematic sensitivity analysis in terms of method, indicator, and dataset selection. Regarding methods, in addition to the DML framework, this study also applied traditional lasso and ridge multiple regression models to directly estimate treatment effects of greenspace on air pollution in a single step. Since lasso and ridge regression use regularization to address multicollinearity among predictors, the Optuna tool was also employed to select optimal regularization hyperparameters before conducting lasso and ridge regression. Since lasso regression shrinks some coefficients to zero, confidence intervals estimated via bootstrapping may result in mean values lying outside the confidence bounds. To address this, we used the median of the coefficients as the representative average. Regarding indicators, first, we compared the treatment effects of greenspace on different air pollutants, including $PM_{2.5}$, PM_1, and PM_{10}. The PM_1 and PM_{10} concentration data were also collected from our mobile monitoring campaign, alongside $PM_{2.5}$ data. Data preprocessing and treatment effect estimation for PM_1 and PM_{10} were consistent with those for $PM_{2.5}$. Second, we compared the treatment effects of different greenspace indicators, including street-view-imagery-based overall greenspace, satellite-based NDVI, and canopy height, on air pollution ($PM_{2.5}$ concentrations). Canopy height data at 1 m resolution for Hong Kong were obtained from the Global and Regional Canopy Height Maps, which were generated using machine learning models on high-resolution Maxar satellite imagery[12]. Notably, canopy height data do not exhibit seasonal variation. In addition, the normalized difference vegetation index (NDVI) was employed as a top-down, two-dimensional measure of street greenspace, enabling further comparison with street view images[13–15]. NDVI data at 10 m resolution were derived from Sentinel-2 satellite imagery via the Google Earth Engine platform, covering the period from November 19, 2021, to April 6, 2023. The imagery was processed using the s2cloudless algorithm to minimize the effects of clouds and shadows[16,17]. Specifically, NDVI is defined as:

$$NDVI = \frac{\rho_{NIR} - \rho_{Red}}{\rho_{NIR} + \rho_{Red}}$$

where ρ_{NIR} and ρ_{Red} represent the surface reflectance values from the near-infrared and red bands, respectively. NDVI theoretically ranges from -1 to 1, with higher values indicating denser vegetation coverage[15]. To mitigate the influence of negative NDVI values, often associated with water bodies and shadows, on measurement accuracy, these values were recoded to zero[14]. Ultimately, the average canopy height and NDVI values within 50 m buffers along streets were used as comparable metrics of greenspace. Notably, NDVI retains seasonal variation, while canopy height is a constant value. To facilitate the indicator comparison, all dependent and independent variables in the second stage of the DML framework were standardized using the robust scaler method. Regarding data, for traffic volume as a covariate, we compared two data sources: the proportion of vehicle types obtained from street view imagery and the absolute counts of vehicles obtained from street detectors, to examine their impacts on the treatment effect estimates from the DML framework. Traffic volume data obtained from street traffic detectors were sourced from the "Traffic Data of Strategic/Major Roads" dataset, provided by the Transport Department[18]. This dataset records information at 30-second intervals, including the number of vehicles, vehicle speeds, and lane details for each traffic detector on major roads. To match our study, we first extracted traffic detector records that corresponded to the research period (formatted as year-month). Next, we aggregated the vehicle counts across all lanes for each traffic detector. Subsequently, we calculated the seasonal averages (spring (December to February), summer (March to May), autumn (June to August), and winter (September to November)) of vehicle counts for each traffic detector. Finally, these seasonal averages were spatially linked to the urban streets defined in this study. This allowed us to calculate the seasonal average vehicle counts for each street, which were then used as a measurement for traffic volume. These traffic volume values were incorporated as control variables in the double machine learning (DML) framework.

Text S11. Feature importance summary when using extreme gradient boosting (XGBoost) models for predicting $PM_{2.5}$, greenspace, tree, mid-level vegetation, and grass in the first stage of DML framework using the seasonal and full-season datasets.

To compare the contributions of different predictors (i.e., covariates) to the prediction of air pollution and greenspace across all XGBoost models, we used mean information gain as the evaluation metric and visualized the feature importance of all XGBoost models (see Supplementary Figures S13–S17). For ease of comparison, the mean information gain values within each model were scaled using min-max normalization to obtain relative feature importance, with values ranging from 0 to 1. We observed that, for the prediction of $PM_{2.5}$, background $PM_{2.5}$, anthropogenic NMVOC emissions, and meteorological factors (including temperature, rainfall, wind speed, and relative humidity) consistently exhibited high contributions (feature importance values) across seasonal datasets (spring, summer, autumn, and winter). In the full-season dataset, in addition to background $PM_{2.5}$, anthropogenic NMVOC emissions, and meteorological factors, seasonal factors also showed significant contributions to $PM_{2.5}$ prediction. For the prediction of overall greenspace, building density, street structure (height-to-width (H/W) ratio), and traffic volume consistently showed high contributions across seasonal datasets (spring, summer, autumn, and winter). In the full-season dataset, these three factors remained highly influential, while seasonal factors also exhibited significant contributions to the prediction of overall greenspace. For the prediction of trees, grass, mid-level vegetation, building density demonstrated high contributions across seasonal and full-season datasets. While in the full-season dataset, the contribution of seasonal factors decreased to a moderate level. For the prediction of mid-level vegetation, grass, trees, and traffic volume exhibited the highest contributions across seasonal and full-season datasets. While in the full-season dataset, the contribution of seasonal factors had a low level. For the prediction of grass, trees and mid-level vegetation consistently exhibited high contributions across both seasonal and full-season datasets. Street structure also showed high contributions (except in winter). While in the full-season dataset, the contribution of seasonal factors

was also at a moderate level. The results of feature importance for predicting trees, mid-level vegetation, and grass revealed that these three vegetation types contribute most significantly to each other's predictions. This suggests a potential synchrony in the distribution of these vegetation types within the urban street environments of Hong Kong.

Text S12. Materials, methods, and results on the influence of greenspace BVOC emissions on its effect on air pollution.

The literature indicates that BVOCs emitted by vegetation can enhance atmospheric oxidative capacity, promote secondary organic aerosol (SOA) formation, and potentially exacerbate particulate pollution[19,20]. To examine the potential relevance of this mechanism in the present study, we incorporated a BVOC proxy into our main model and further assessed its interaction with the greenspace variables.

The BVOC data used in this study were derived from VOC speciation measurements collected at seven air quality monitoring stations operated by the Hong Kong SAR government[21] (Figures S21-22). It should be noted that these stations provide observations of ambient VOC species concentrations (ppb) rather than direct emission estimates. Given the lack of high-resolution street-level BVOC emission data, together with the limited number and sparse spatial distribution of existing monitoring stations, these observations are insufficient to characterize BVOC variability across all streets in Hong Kong. Accordingly, we treat this analysis as a supplementary experiment only.

Specifically, for each monitoring station, we matched the 10 nearest streets by spatial distance and used the station-level observations as an approximate representation of BVOC conditions for those streets. In terms of indicator selection, this study used isoprene as a proxy for BVOC levels. Isoprene is one of the most important markers of vegetation-derived BVOCs and is widely used as a representative compound in the relevant literature[19,20]. Based on the monitoring station data, we calculated the seasonal mean isoprene concentrations from 2023 to 2024 and matched these values to the corresponding streets. We then estimated the average treatment effect (ATE) and heterogeneous effects using a DML model with the xgb-ols specification (i.e., XGBoost used in the first stage and ordinary least squares used in the second stage), treating BVOC as a moderating variable. The results are presented in Table S5.

The results show that the BVOC proxy significantly and positively moderated the effects of greenspace, trees, and mid-level vegetation. This finding suggests that under conditions of higher BVOC levels, the air-pollution mitigation effect of vegetation was weakened. The result is broadly consistent with previous studies indicating that BVOCs may offset part of the air quality benefits of vegetation by enhancing atmospheric oxidation and promoting SOA formation[19,20]. However, because this supplementary analysis relies on the spatial approximation of BVOC exposure based on a small number of monitoring stations, both sample representativeness and estimation precision are limited. The results should therefore be interpreted with caution and regarded only as supplementary evidence.

Supporting Information Figures

Figure S1. The study area comprises 1,746 Large Subunit Groups (LSUGs) and 18 District Council Districts (DCDs) in Hong Kong. Panel A shows the spatial distribution of LSUGs and DCDs and locates Hong Kong relative to mainland China. Panels B and C provide detailed examples from Sha Tin Center Street and New Praya in Kennedy Town, respectively, illustrating the streetscape, road network, and building footprints. Panel D demonstrates the application of the area dominance method based on the Urban Development Intensity indicator (UDI) indicator and provides the full names of the 18 DCDs. Publicly available boundary data was provided by the Hong Kong SAR Government.

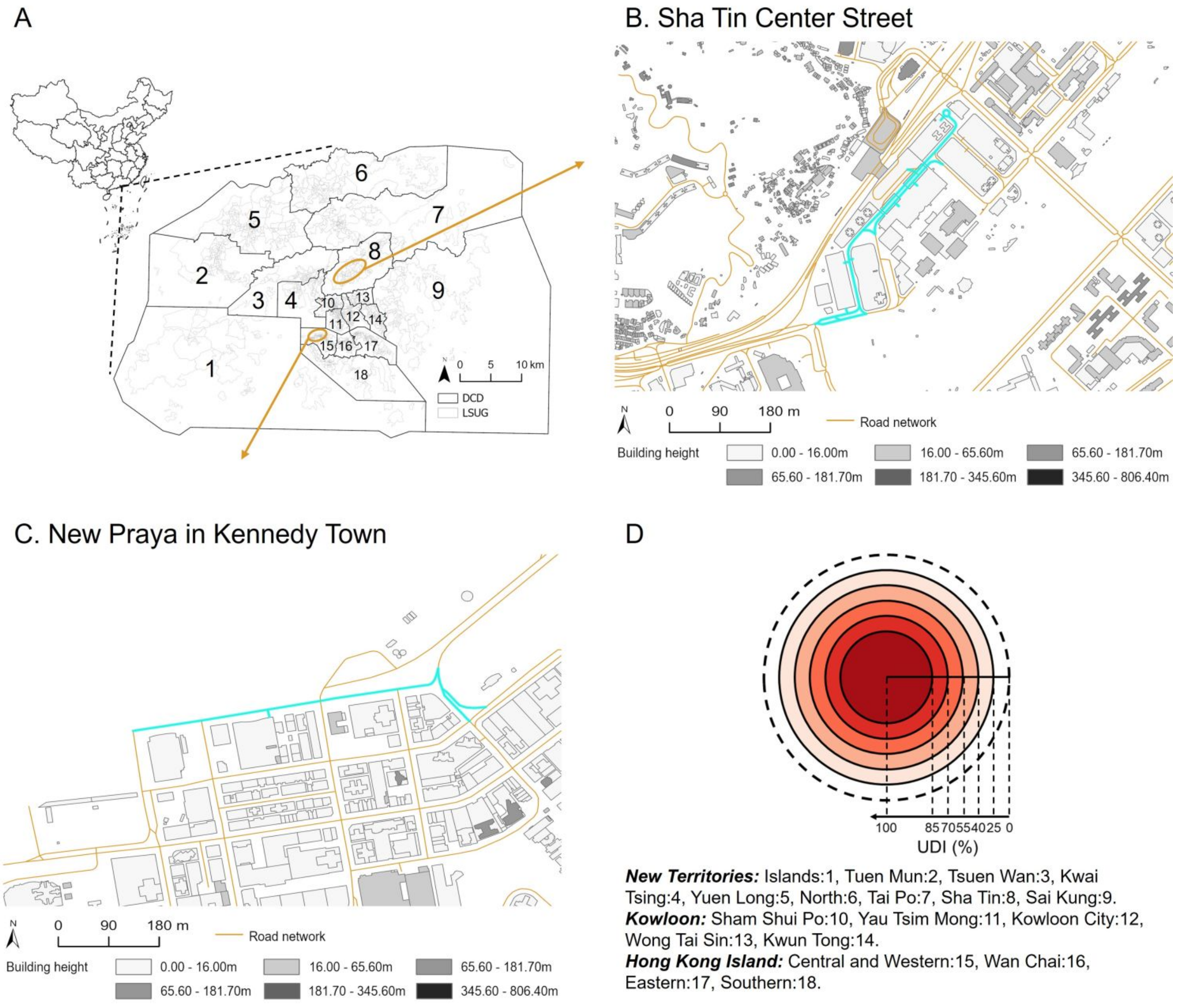

Figure S2. Spatial distribution of $PM_{2.5}$ concentrations across four seasons in urban areas of Hong Kong. Publicly available boundary data was provided by the Hong Kong SAR Government.

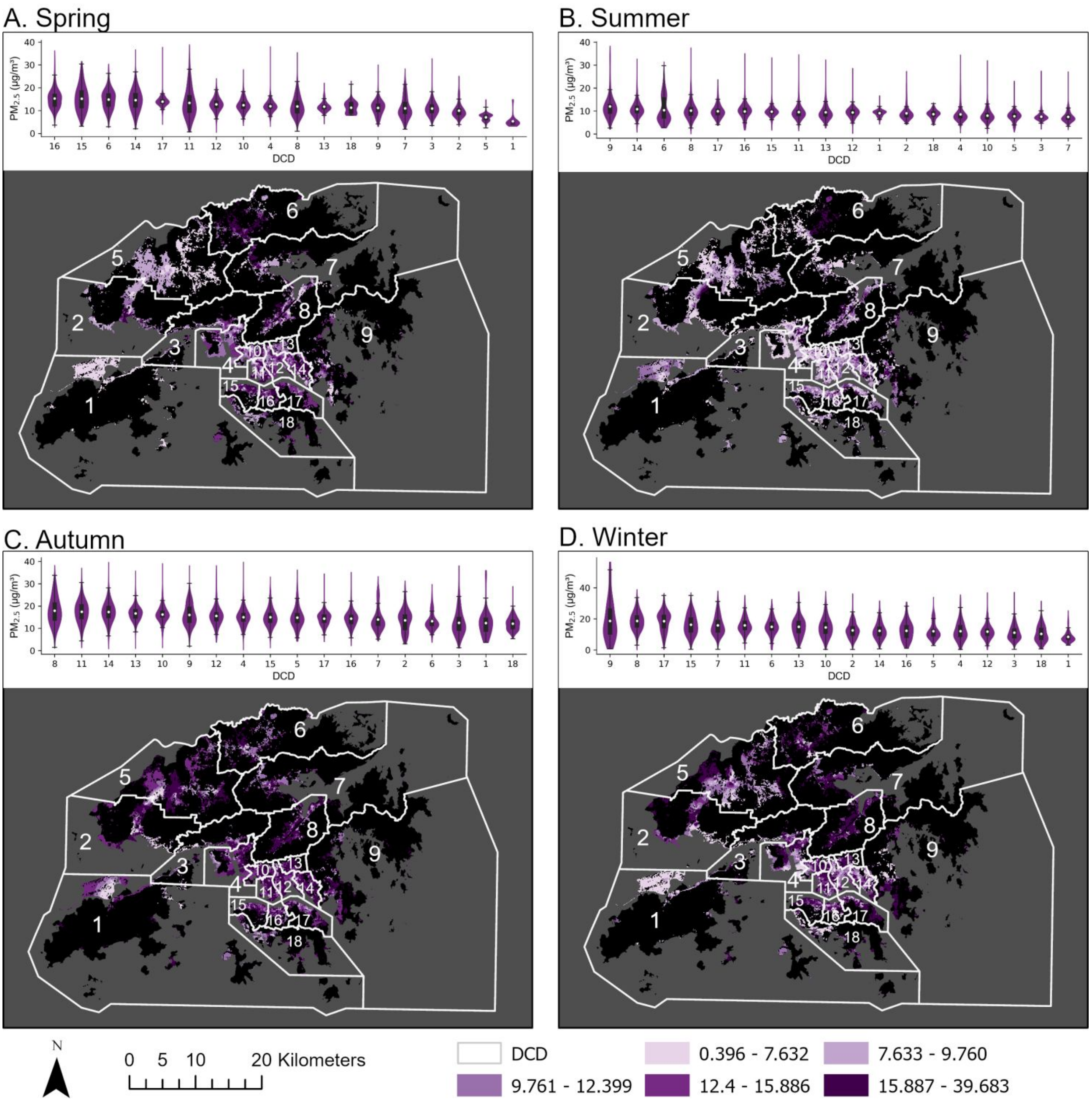

Figure S3. Spatial distribution of street greenspace across four seasons in urban areas of Hong Kong. Publicly available boundary data was provided by the Hong Kong SAR Government.

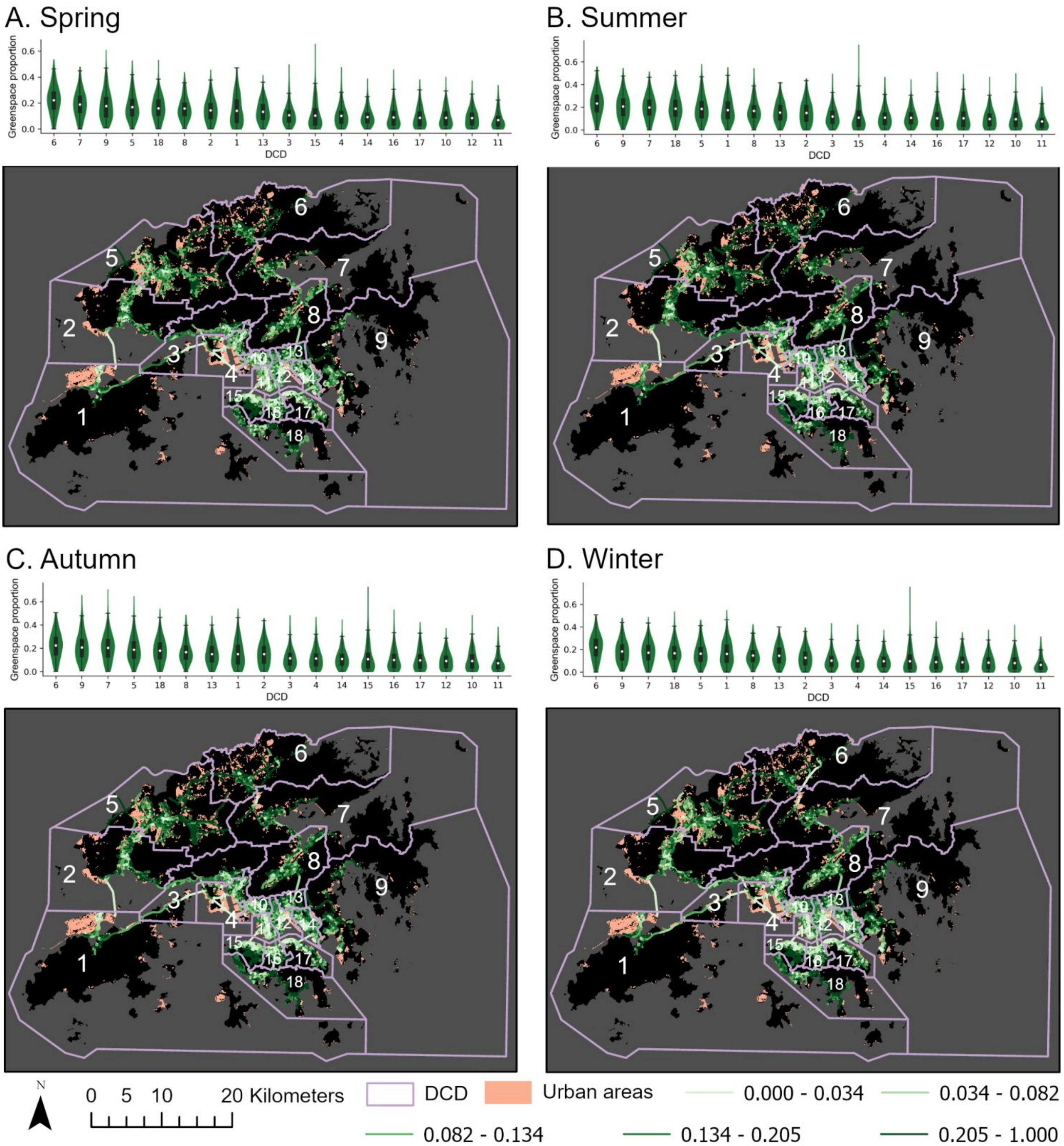

Figure S4. Spatial distribution of street trees across four seasons in urban areas of Hong Kong. Publicly available boundary data was provided by the Hong Kong SAR Government.

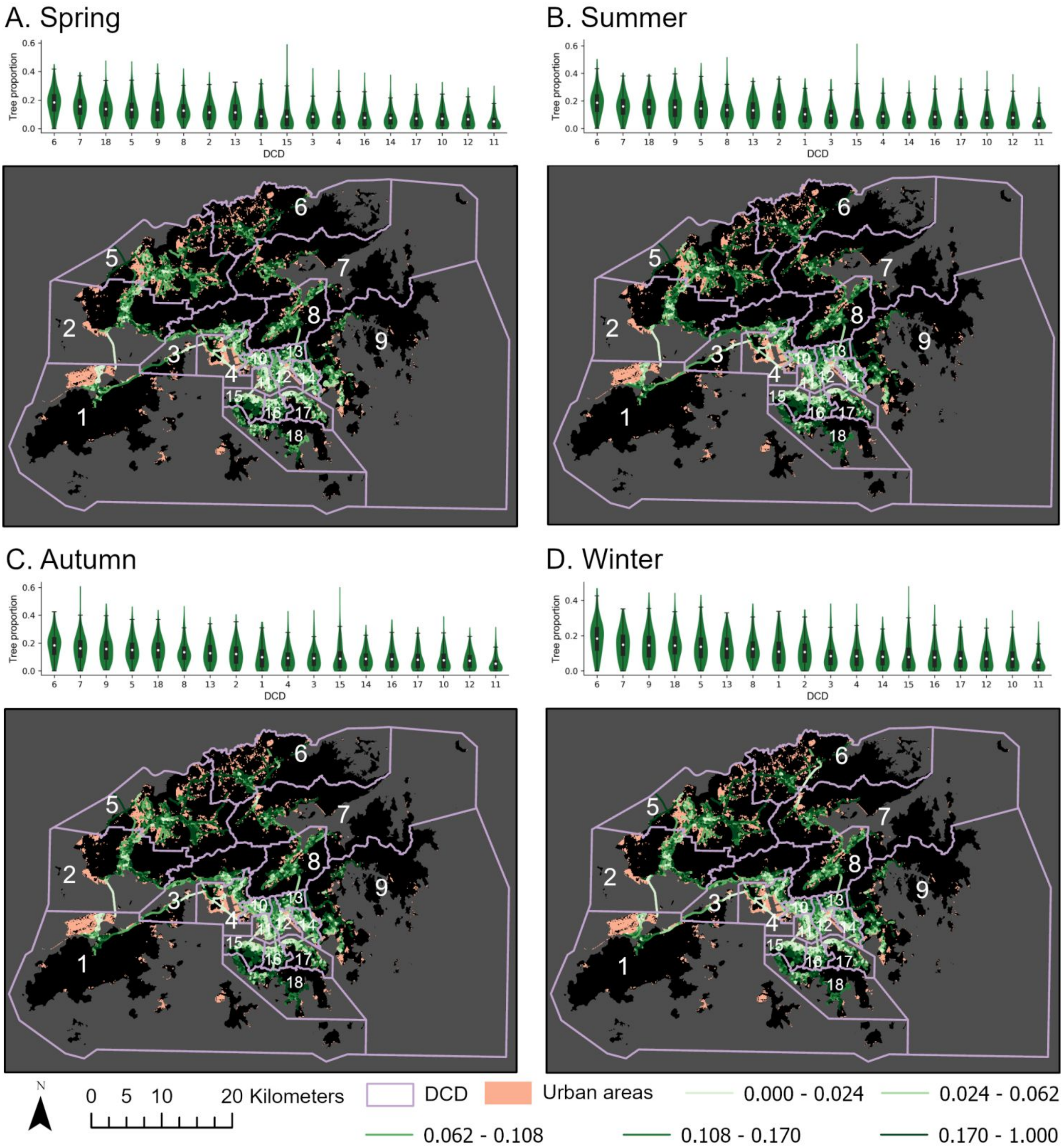

Figure S5. Spatial distribution of street mid-level vegetation across four seasons in urban areas of Hong Kong. Publicly available boundary data was provided by the Hong Kong SAR Government.

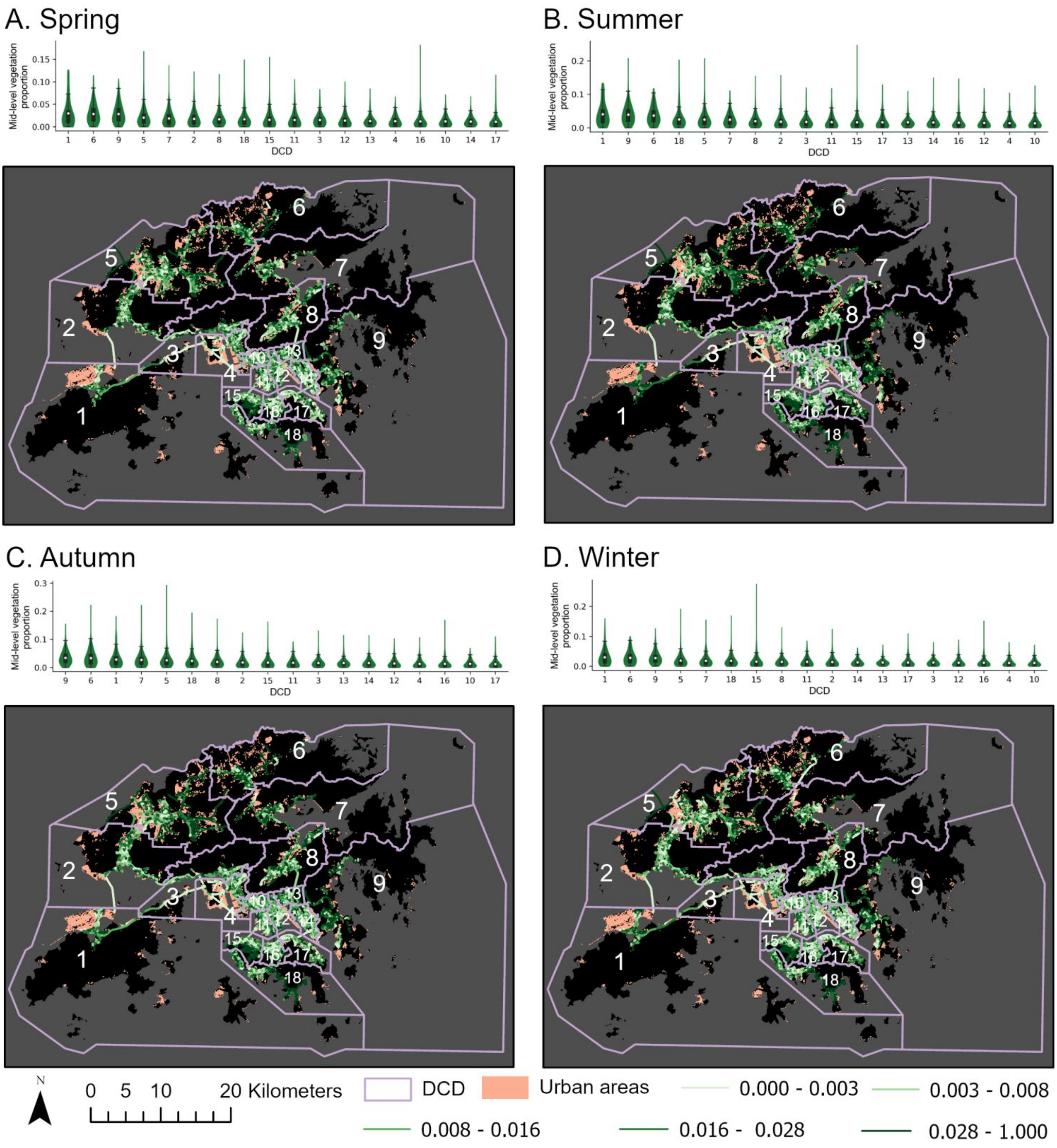

Figure S6. Spatial distribution of street grass across four seasons in urban areas of Hong Kong. Publicly available boundary data was provided by the Hong Kong SAR Government.

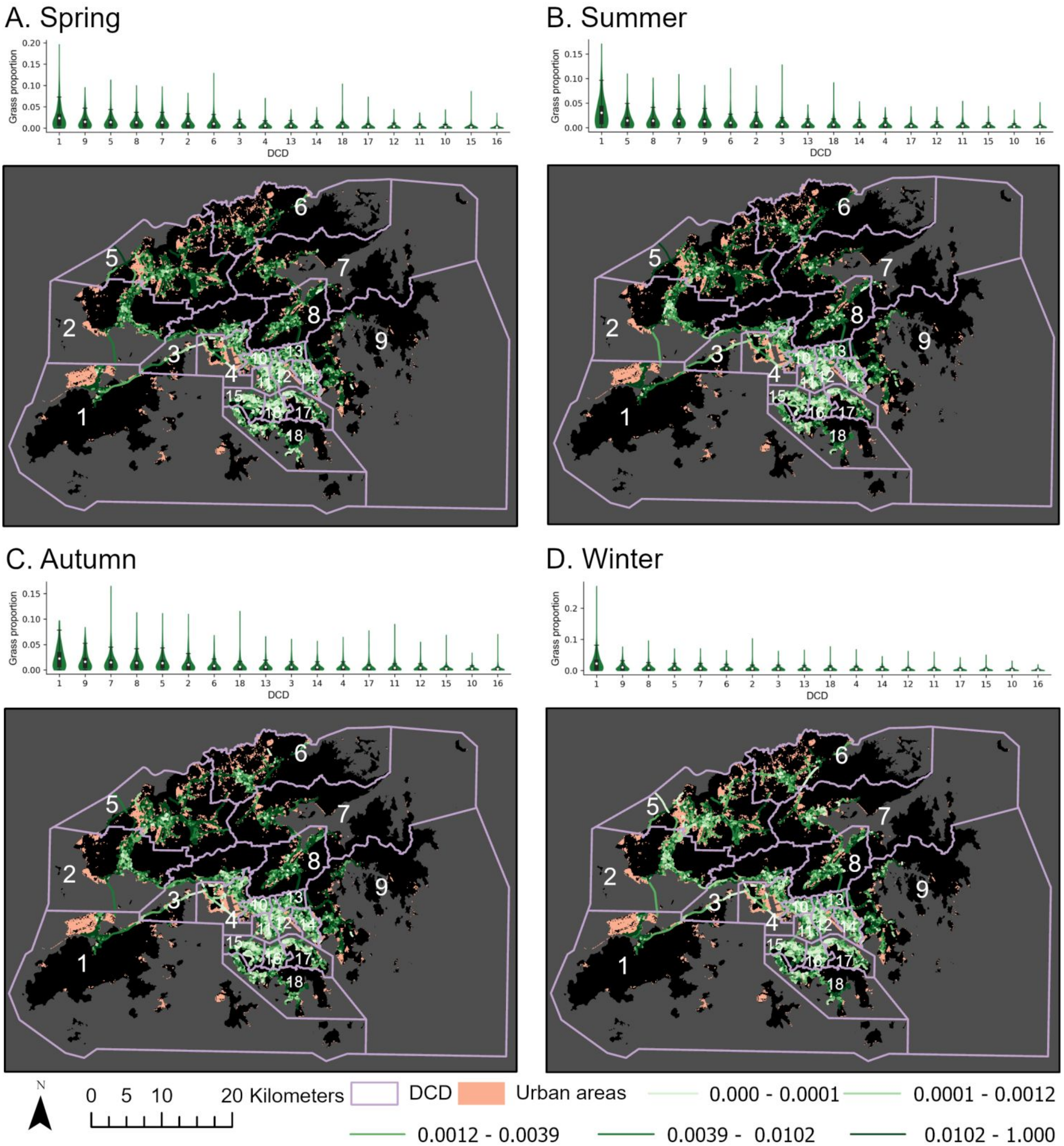

Figure S7. Schematic of street structure calculation based on average building height and street width, with a 50 m limitation on single-side street width.

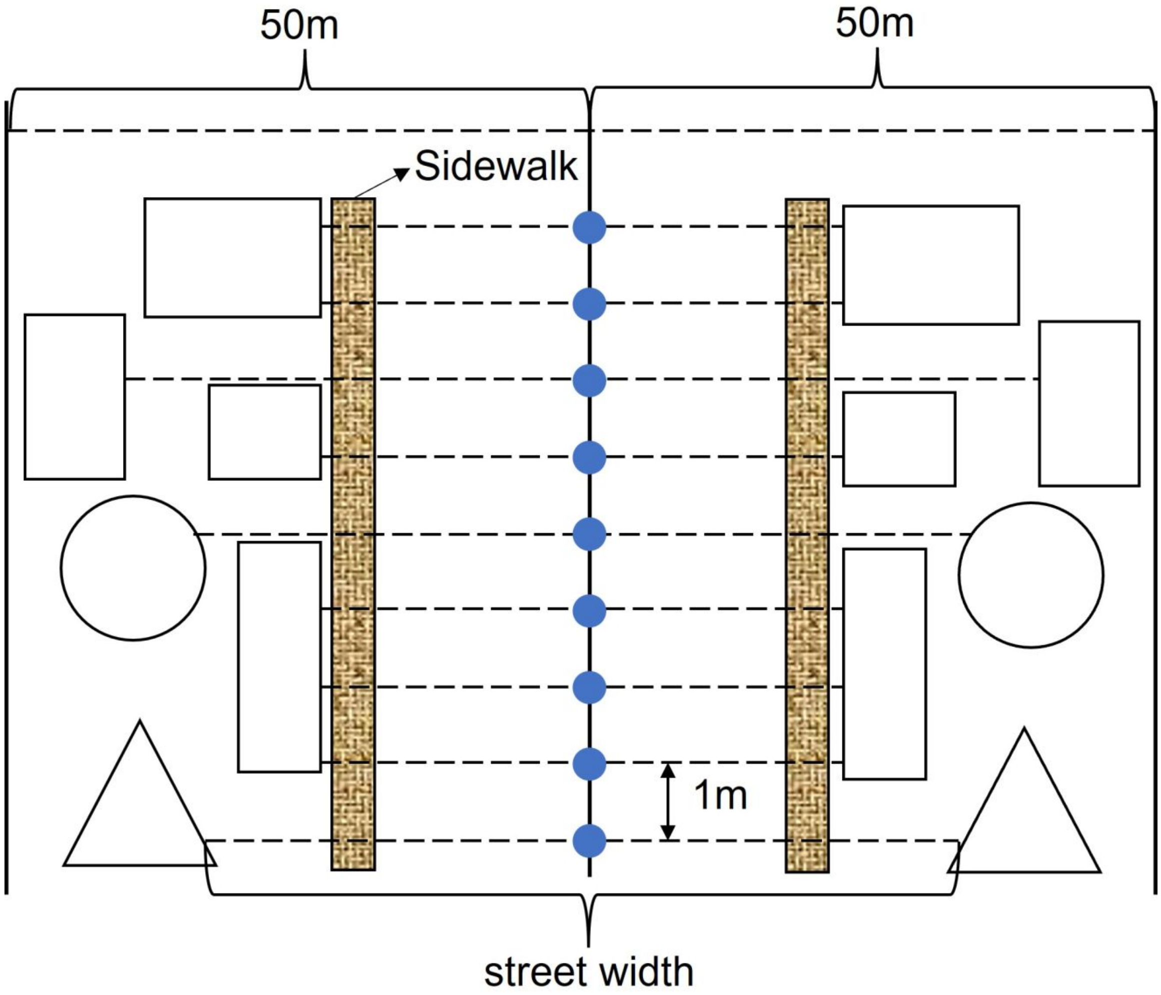

Figure S8. The temporal changes of daily records from fixed monitoring stations for background $PM_{2.5}$ (A), background PM_{10} (B), rainfall (C), relative humidity (D), temperature (E), wind speed (F), and traffic volume (G).

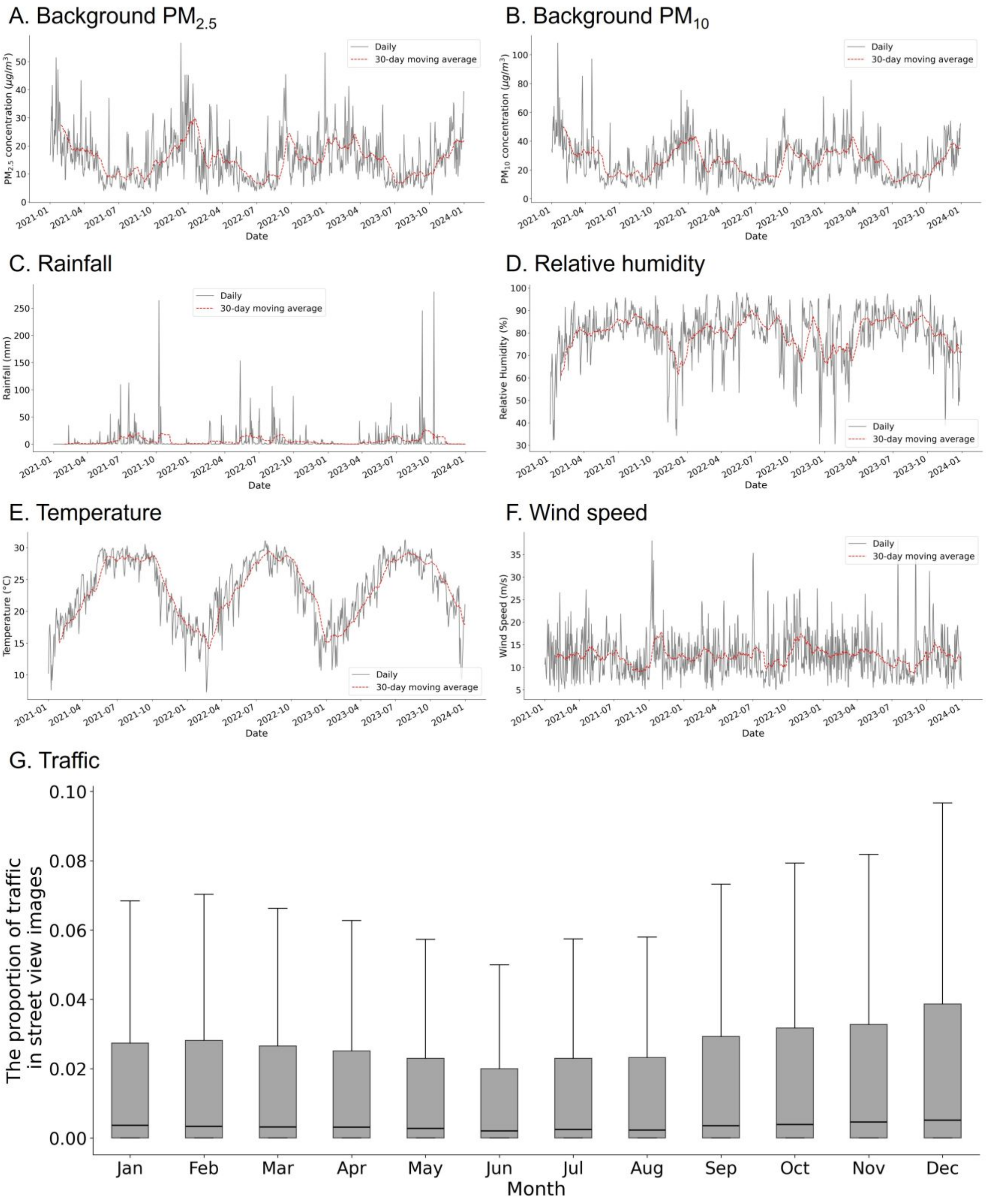

Figure S9. Model performance comparison for $PM_{2.5}$, greenspace, tree, mid-level vegetation, and grass in stage one of double machine learning using data in ***spring***. The model performance is measured by the root mean squared error (RMSE), where a smaller value indicates better model performance. The candidate models for comparison are lasso regression (lasso), ridge regression (ridge), decision trees (dt), random forest (rf), gradient boosted decision trees (gbdt), light gradient boosting machine (lgbm), and extreme gradient boosting (xgb).

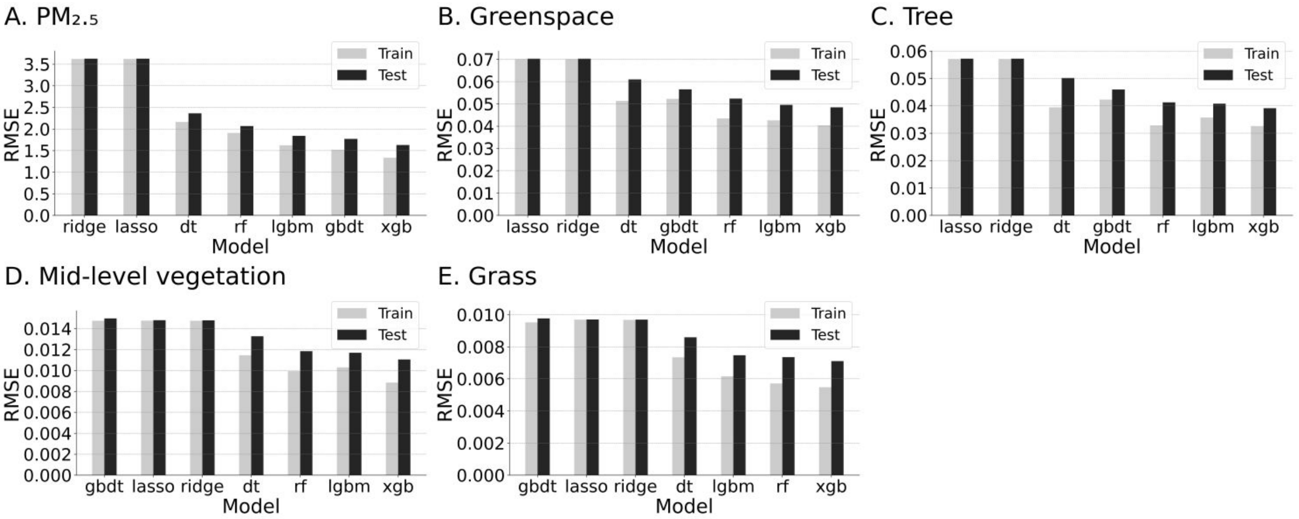

Figure S10. Model performance comparison for $PM_{2.5}$, greenspace, tree, mid-level vegetation, and grass in stage one of double machine learning using data in ***summer***. The model performance is measured by the root mean squared error (RMSE), where a smaller value indicates better model performance. The candidate models for comparison are lasso regression (lasso), ridge regression (ridge), decision trees (dt), random forest (rf), gradient boosted decision trees (gbdt), light gradient boosting machine (lgbm), and extreme gradient boosting (xgb).

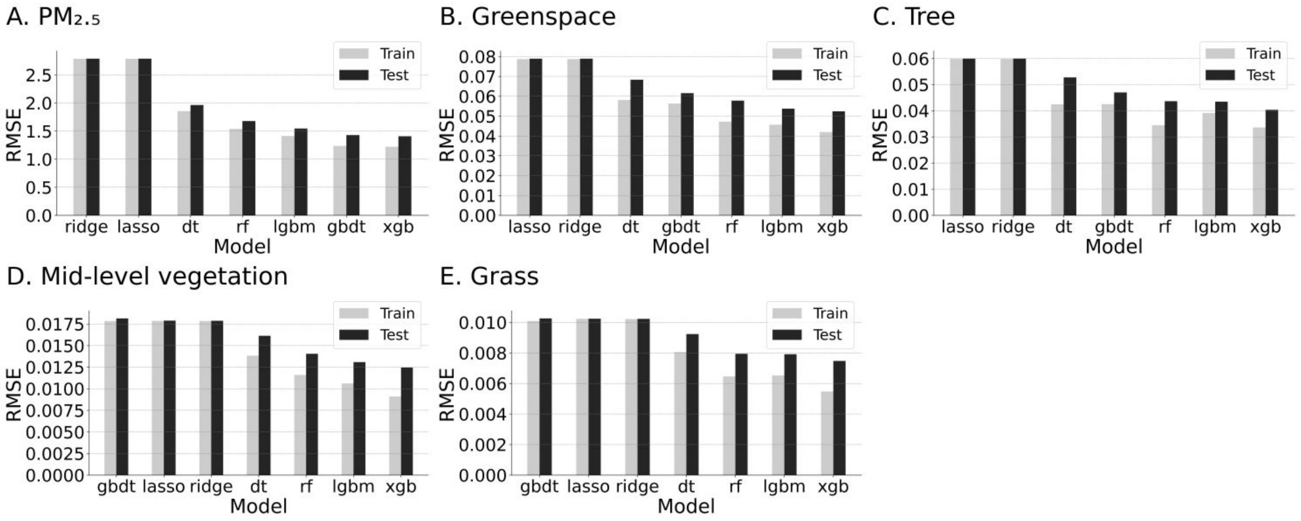

Figure S11. Model performance comparison for $PM_{2.5}$, greenspace, tree, mid-level vegetation, and grass in stage one of double machine learning using data in ***autumn***. The model performance is measured by the root mean squared error (RMSE), where a smaller value indicates better model performance. The candidate models for comparison are lasso regression (lasso), ridge regression (ridge), decision trees (dt), random forest (rf), gradient boosted decision trees (gbdt), light gradient boosting machine (lgbm), and extreme gradient boosting (xgb).

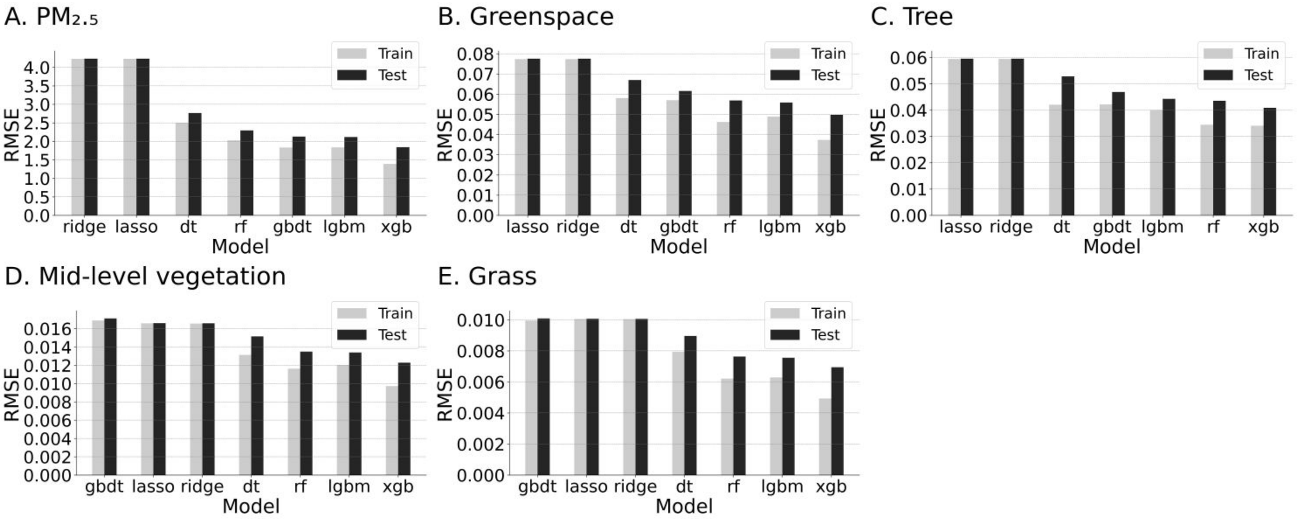

Figure S12. Model performance comparison for $PM_{2.5}$, greenspace, tree, mid-level vegetation, and grass in stage one of double machine learning using data in ***winter***. The model performance is measured by the root mean squared error (RMSE), where a smaller value indicates better model performance. The candidate models for comparison are lasso regression (lasso), ridge regression (ridge), decision trees (dt), random forest (rf), gradient boosted decision trees (gbdt), light gradient boosting machine (lgbm), and extreme gradient boosting (xgb).

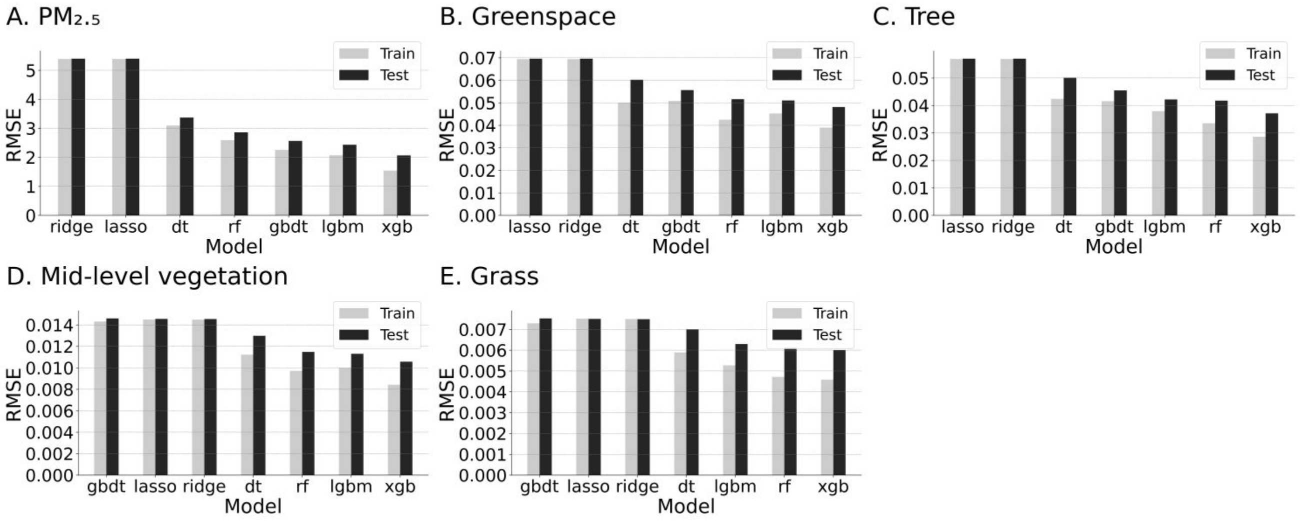

Figure S13. Feature importance when using extreme gradient boosting (XGBoost) model for predicting $PM_{2.5}$ (A), greenspace (B), tree (C), mid-level vegetation (D), and grass (E) in stage one of double machine learning using data from ***all seasons***.

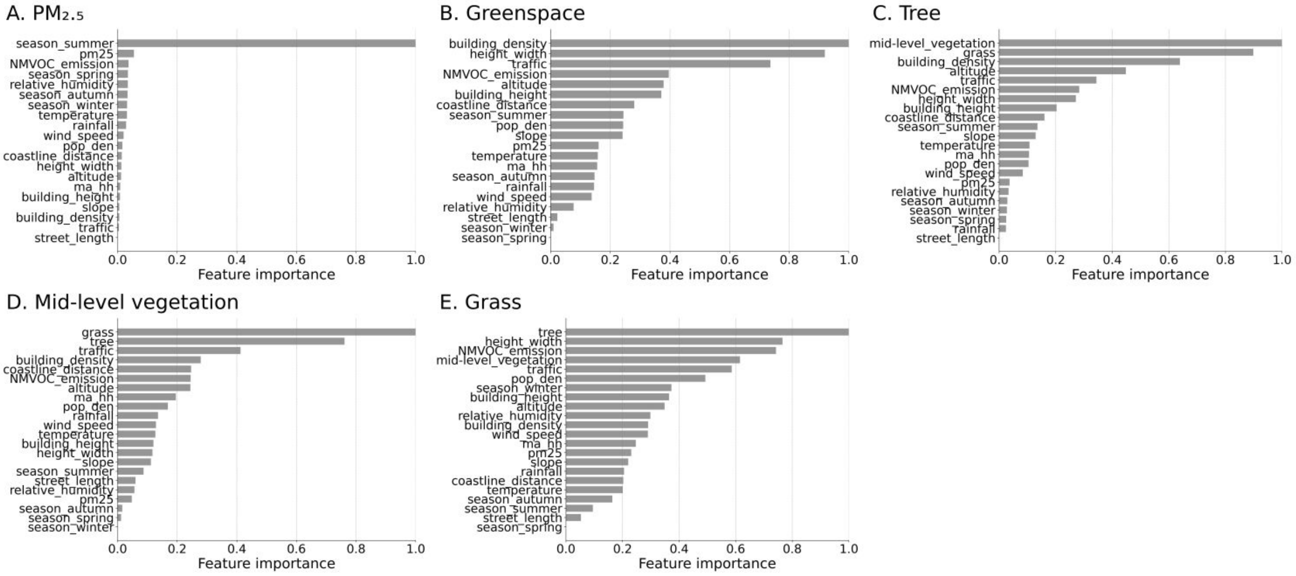

Figure S14. Feature importance when using extreme gradient boosting (XGBoost) model for predicting $PM_{2.5}$ (A), greenspace (B), tree (C), mid-level vegetation (D), and grass (E) in stage one of double machine learning using data in ***spring***.

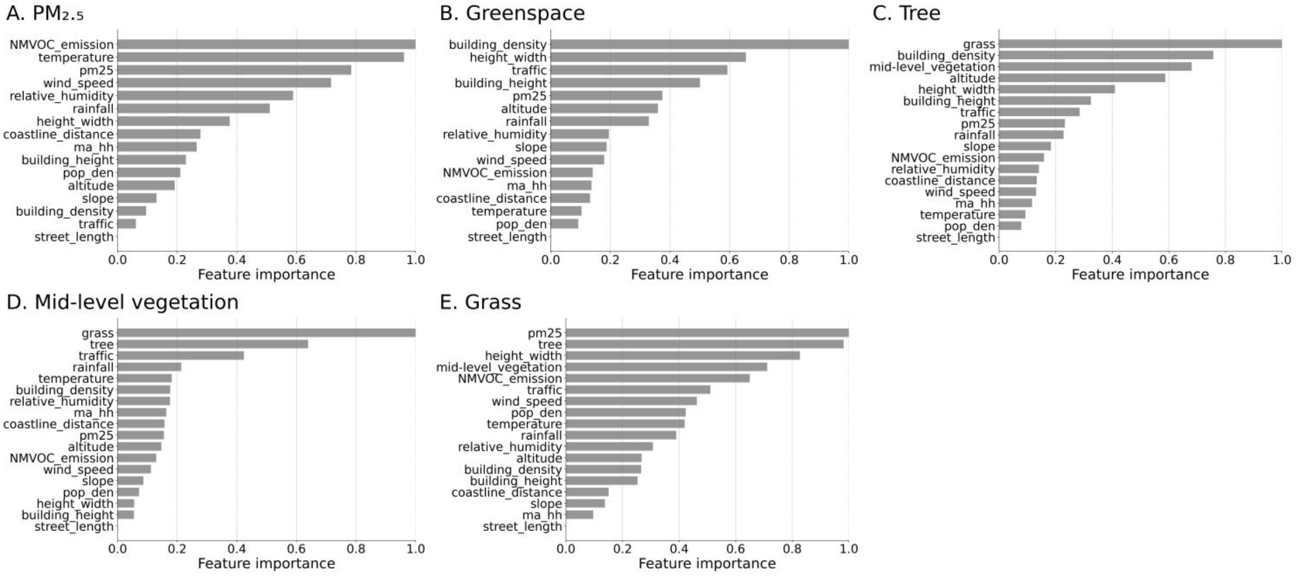

Figure S15. Feature importance when using extreme gradient boosting (XGBoost) model for predicting $PM_{2.5}$ (A), greenspace (B), tree (C), mid-level vegetation (D), and grass (E) in stage one of double machine learning using data in ***summer***.

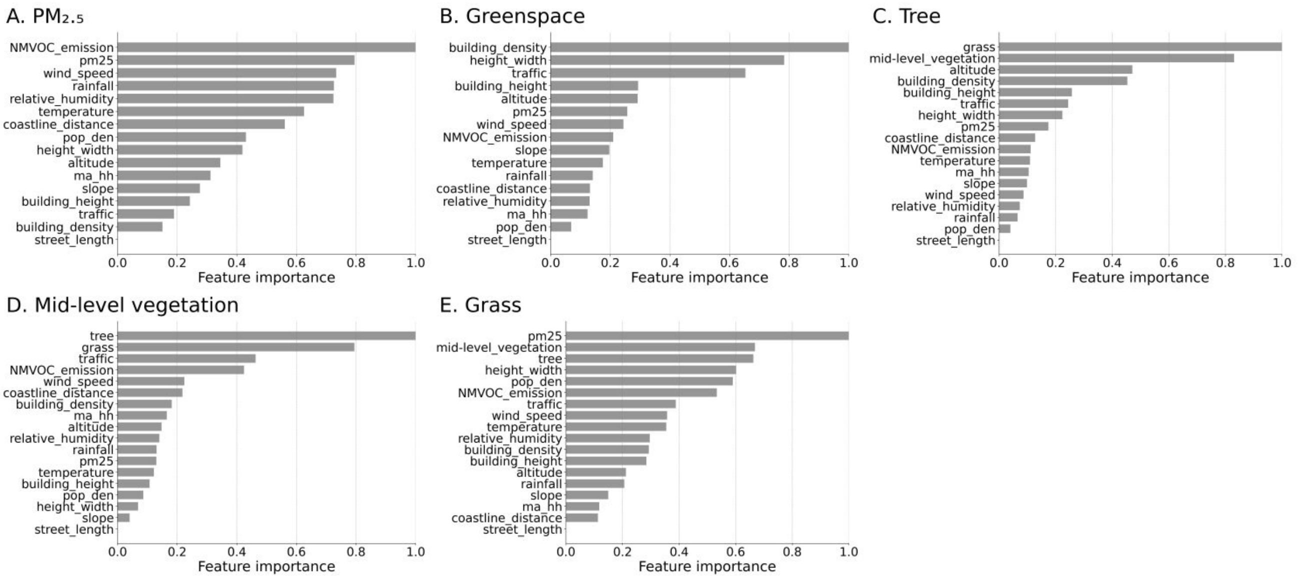

Figure S16. Feature importance when using extreme gradient boosting (XGBoost) model for predicting $PM_{2.5}$ (A), greenspace (B), tree (C), mid-level vegetation (D), and grass (E) in stage one of double machine learning using data in ***autumn***.

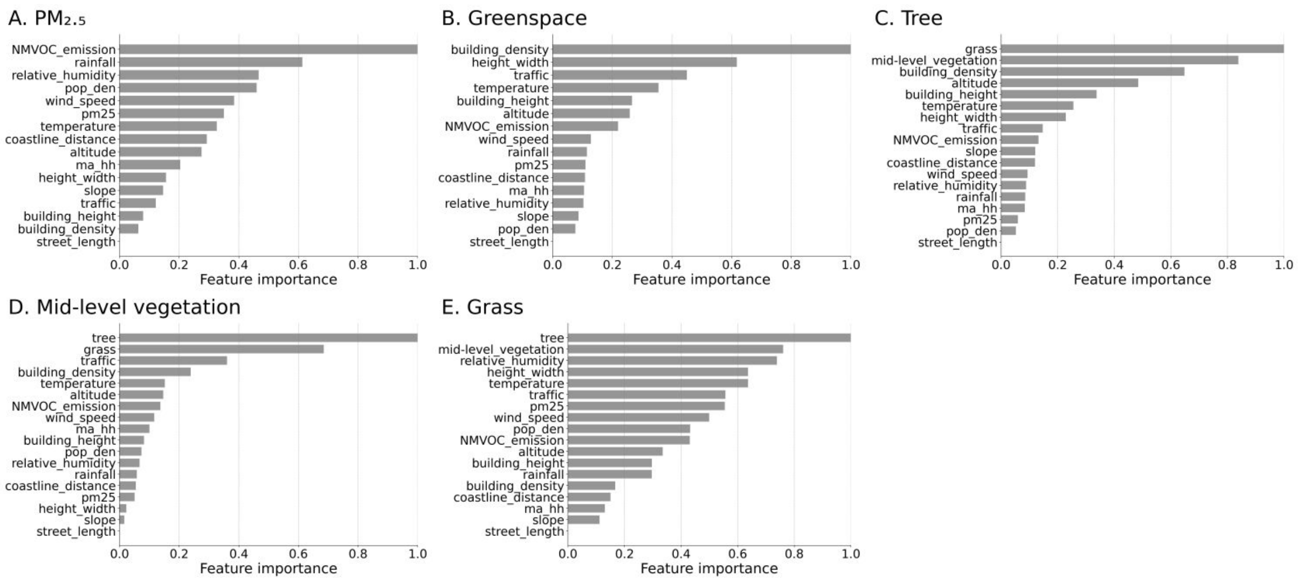

Figure S17. Feature importance when using the extreme gradient boosting (XGBoost) model for predicting $PM_{2.5}$ (A), greenspace (B), tree (C), mid-level vegetation (D), and grass (E) in stage one of double machine learning using data in ***winter***.

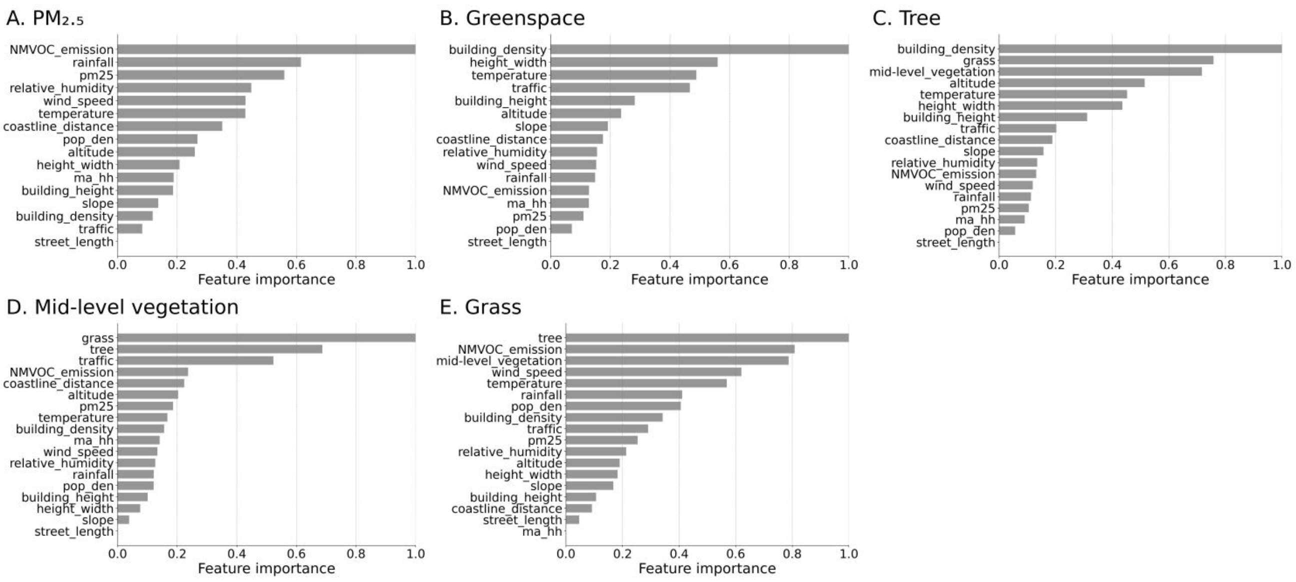

Figure S18. Comparison of average treatment effects (ATEs) of the street view-derived greenspace indicators (overall greenspace, tree, mid-level vegetation, and grass) on air pollutants (PM_1, $PM_{2.5}$, and PM_{10}) using seasonal and full-season datasets.

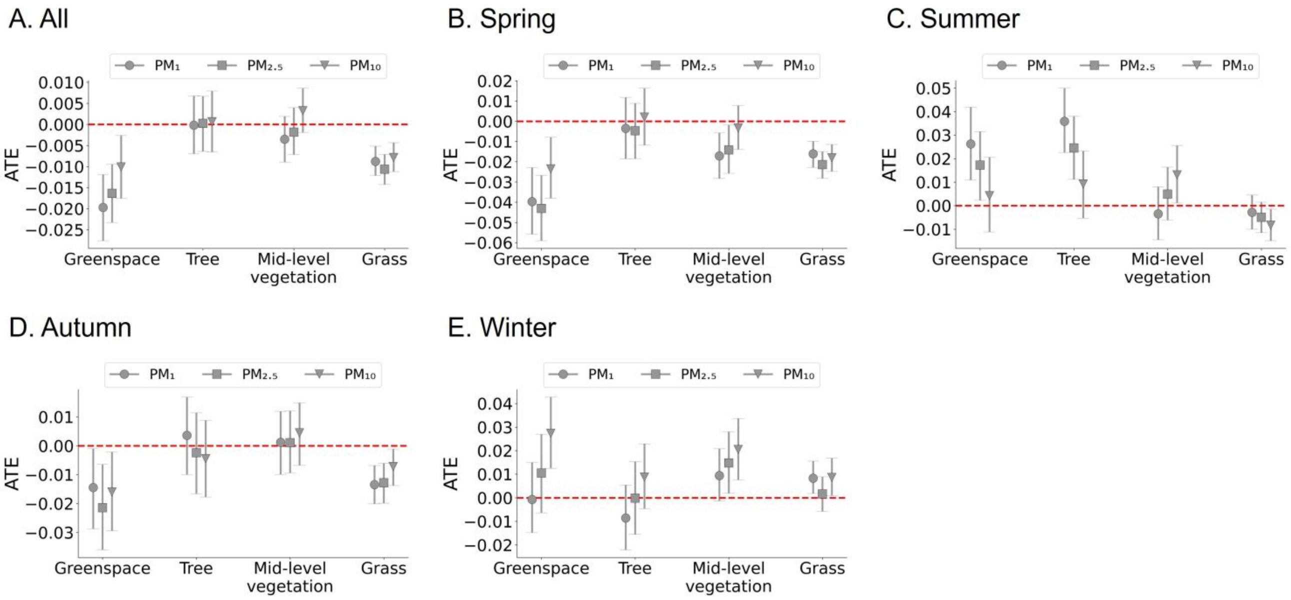

Figure S19. Comparison of average treatment effects (ATEs) of greenspace indicators (greenspace derived from street view images (SVI), NDVI, and canopy height) on $PM_{2.5}$ pollution.

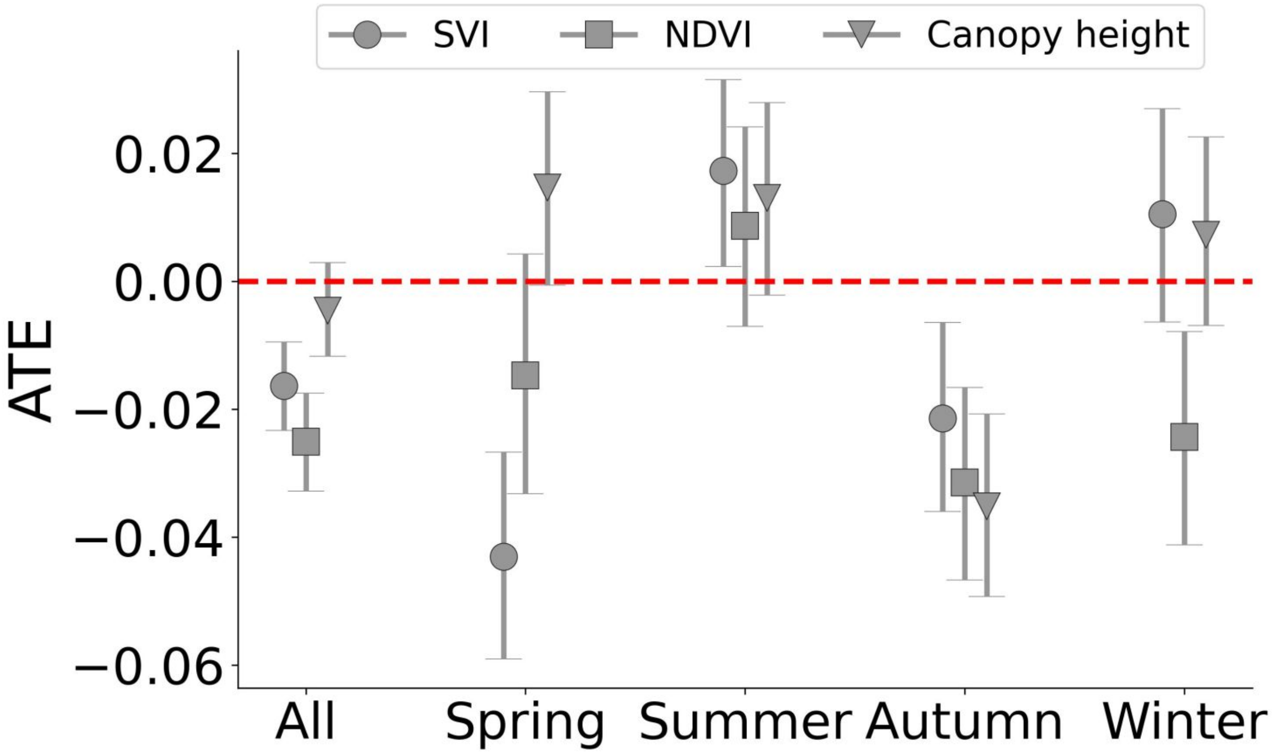

Figure S20. ATE comparison for greenspace, tree, mid-level vegetation, and grass between model 1, model 2, and model 3 in full-season, spring, summer, autumn, and winter. Model 1 uses the traffic volume data derived from street view images across all streets; Model 2 also uses the traffic volume data derived from street view images, while matching streets with street detectors; Model 3 uses the traffic volume data derived from street detectors in limited streets.

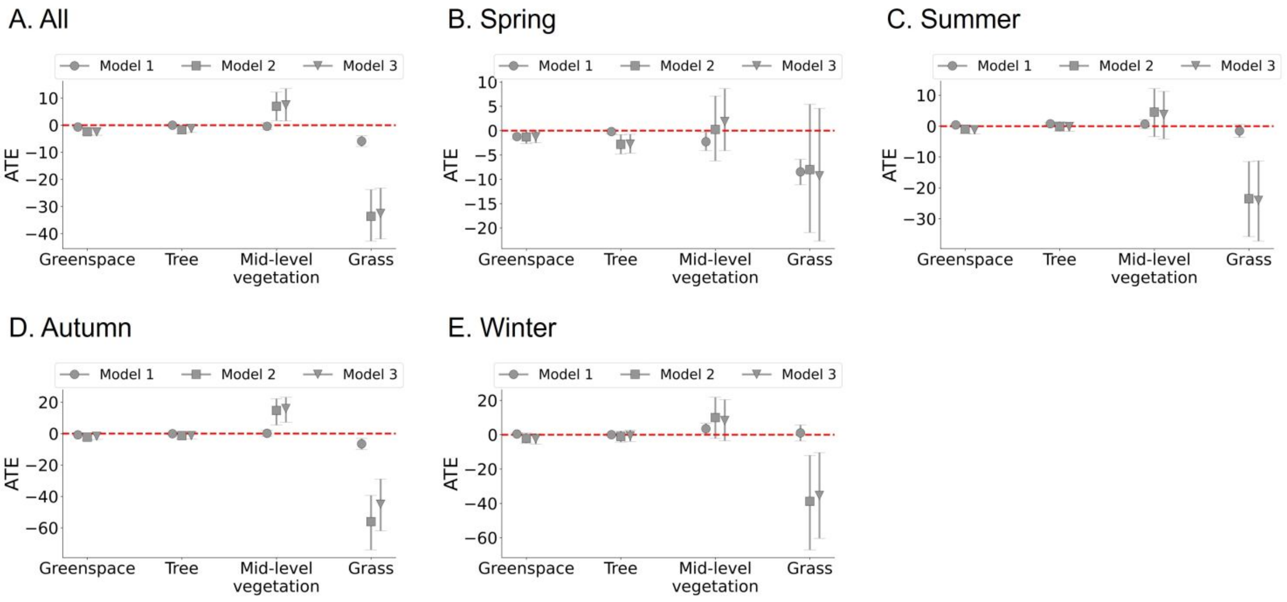

Figure S21. The distribution of government monitoring stations for BVOC emission monitoring. Publicly available boundary data was provided by the Hong Kong SAR Government.

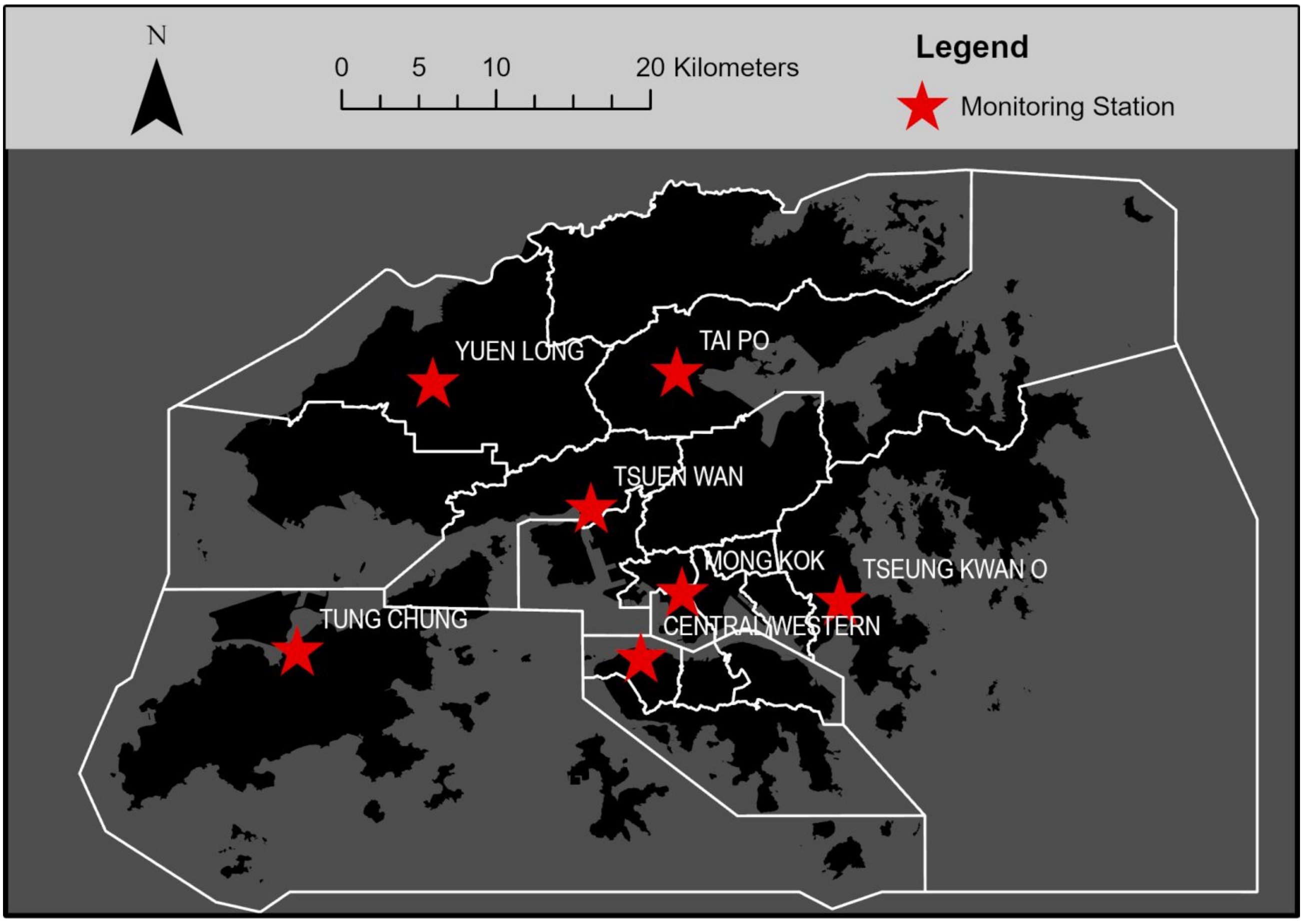

Figure S22. BVOC (using the isoprene as proxy) levels averaged by seasons between 2023 and 2024 derived from government monitoring stations.

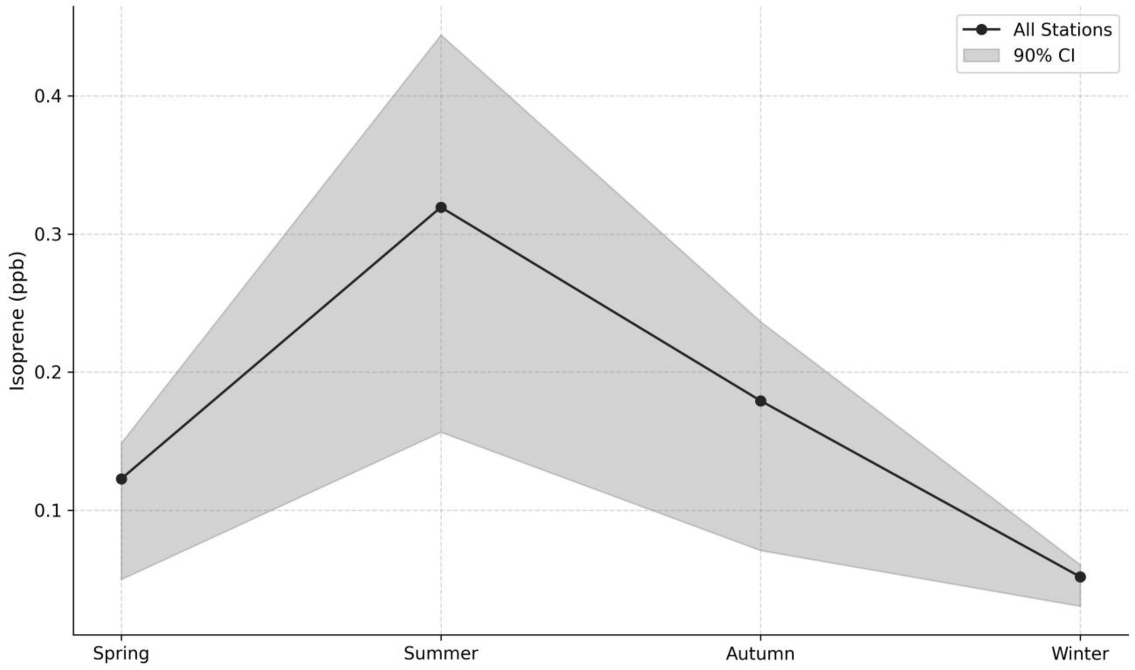

Figure S23. Wind speed averaged by seasons at study streets.

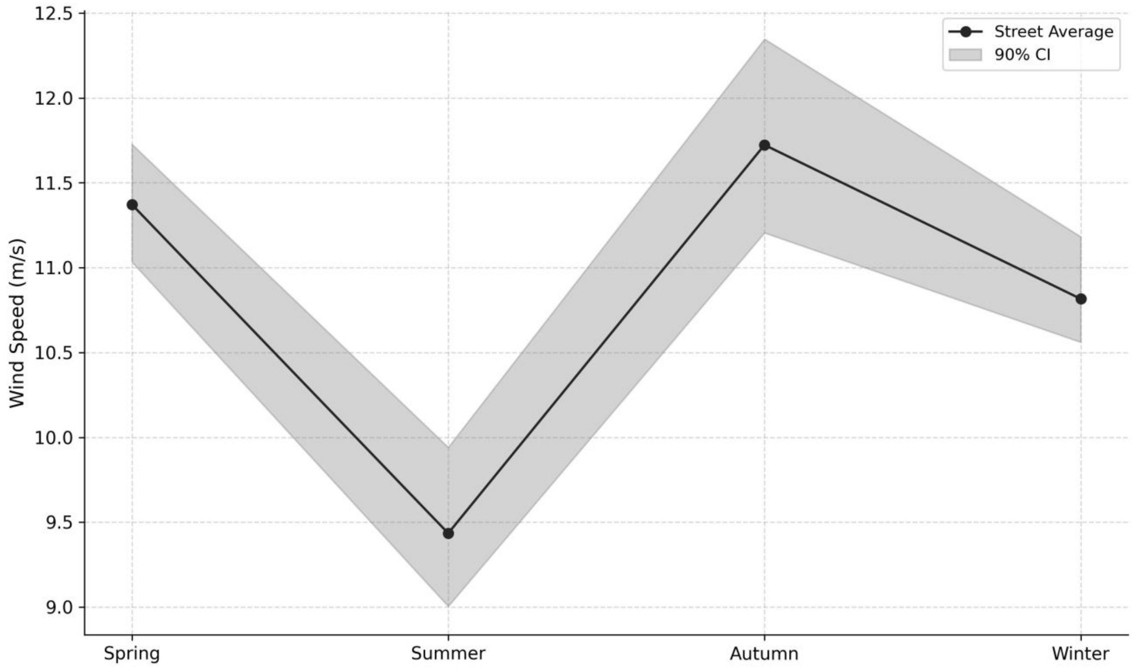

Supporting Information Tables

Table S1. Descriptive results of mobile and static $PM_{2.5}$ air pollution, street greenspace, trees, mid-level vegetation, and grass in urban areas of Hong Kong*.

Indicator	Spring	Summer	Autumn	Winter
Mobile_EBK# ($\mu g/m^3$)	10.730 (4.390)	9.110 (3.218)	14.711 (4.836)	13.439 (6.439)
Static_EBK## ($\mu g/m^3$)	15.434 (0.180)	8.762 (0.040)	15.856 (0.149)	21.132 (0.614)
Street greenspace	0.119 (0.092)	0.133 (0.101)	0.130 (0.100)	0.118 (0.091)
Street trees	0.096 (0.079)	0.106 (0.084)	0.105 (0.084)	0.097 (0.079)
Street mid-level vegetation	0.016 (0.017)	0.020 (0.021)	0.019 (0.020)	0.016 (0.017)
Street grass	0.007 (0.011)	0.007 (0.012)	0.007 (0.012)	0.005 (0.009)

* The table presents the seasonal means for each indicator, with standard deviations provided in parentheses.

#: Mobile_EBK denotes the simulated air pollution derived from mobile air pollution monitoring data using the empirical Bayesian kriging (EBK) method.

##: Static_EBK denotes the simulated urban air pollution derived from static air pollution monitoring data using the empirical Bayesian kriging (EBK) method.

Table S2. The estimated average treatment effects (ATEs) of overall greenspace, trees, mid-level vegetation, and grass on $PM_{2.5}$ concentrations across different seasons, using different models. The The models, xgb-ols, lasso-ols, ridge-ols, dt-ols, rf-ols, gbdt-ols, and lgbm-ols represent ATE estimates obtained through DML framework. In the first stage, each model employs its respective algorithm: extreme gradient boosting (xgb), lasso regression (lasso), ridge regression (ridge), decision trees (dt), random forest (rf), gradient boosted decision trees (gbdt), and light gradient boosting machine (lgbm) model. The second stage uniformly applies an ordinary least squares (ols) model with bootstrap techniques. The models, lasso and ridge represent ATE estimates obtained through traditional lasso and ridge regressions.

Model	Season	Treatment	Mean	Low CI	Upper CI
xgb-ols	All	Greenspace	-0.641	-0.913	-0.372
xgb-ols	Spring	Greenspace	-1.219	-1.672	-0.757
xgb-ols	Summer	Greenspace	0.390	0.051	0.713
xgb-ols	Autumn	Greenspace	-0.751	-1.260	-0.226
xgb-ols	Winter	Greenspace	0.408	-0.250	1.050
xgb-ols	All	Tree	0.014	-0.379	0.395
xgb-ols	Spring	Tree	-0.174	-0.690	0.331
xgb-ols	Summer	Tree	0.762	0.347	1.178
xgb-ols	Autumn	Tree	-0.107	-0.732	0.501
xgb-ols	Winter	Tree	-0.006	-0.854	0.841
xgb-ols	All	Mid-level vegetation	-0.408	-1.593	0.879
xgb-ols	Spring	Mid-level vegetation	-2.275	-4.132	-0.298
xgb-ols	Summer	Mid-level vegetation	0.633	-0.789	2.108
xgb-ols	Autumn	Mid-level vegetation	0.183	-1.683	2.159
xgb-ols	Winter	Mid-level vegetation	3.480	0.457	6.596
xgb-ols	All	Grass	-5.890	-7.893	-3.940
xgb-ols	Spring	Grass	-8.464	-11.142	-5.911
xgb-ols	Summer	Grass	-1.565	-3.659	0.472
xgb-ols	Autumn	Grass	-6.506	-10.098	-3.075
xgb-ols	Winter	Grass	1.055	-3.721	5.696
lasso	All	Greenspace	-2.432	-2.784	-2.111

lasso	Spring	Greenspace	-1.404	-1.975	-0.823
lasso	Summer	Greenspace	-0.762	-1.179	-0.346
lasso	Autumn	Greenspace	-2.460	-3.105	-1.825
lasso	Winter	Greenspace	-3.557	-4.517	-2.586
lasso	All	Tree	-1.431	-1.853	-1.032
lasso	Spring	Tree	0.000	0.000	0.000
lasso	Summer	Tree	-0.844	-1.324	-0.367
lasso	Autumn	Tree	-2.015	-2.827	-1.227
lasso	Winter	Tree	-3.454	-4.526	-2.320
lasso	All	Mid-level vegetation	0.000	0.000	0.000
lasso	Spring	Mid-level vegetation	-6.715	-9.539	-4.043
lasso	Summer	Mid-level vegetation	0.000	0.000	0.000
lasso	Autumn	Mid-level vegetation	0.000	0.000	0.000
lasso	Winter	Mid-level vegetation	0.000	0.000	4.117
lasso	All	Grass	-26.000	-28.904	-23.268
lasso	Spring	Grass	-30.121	-34.056	-26.098
lasso	Summer	Grass	-4.557	-7.554	-1.727
lasso	Autumn	Grass	-16.642	-22.226	-10.813
lasso	Winter	Grass	-25.044	-33.569	-15.912
ridge	All	Greenspace	-3.841	-4.175	-3.497
ridge	Spring	Greenspace	-2.382	-2.979	-1.796
ridge	Summer	Greenspace	-1.263	-1.664	-0.829
ridge	Autumn	Greenspace	-3.022	-3.686	-2.346
ridge	Winter	Greenspace	-4.267	-5.236	-3.330
ridge	All	Tree	-3.318	-3.756	-2.876
ridge	Spring	Tree	0.374	-0.362	1.081
ridge	Summer	Tree	-2.142	-2.686	-1.568
ridge	Autumn	Tree	-2.969	-3.843	-2.096
ridge	Winter	Tree	-4.396	-5.573	-3.214
ridge	All	Mid-level vegetation	2.755	1.178	4.337
ridge	Spring	Mid-level vegetation	-9.904	-12.606	-7.210
ridge	Summer	Mid-level vegetation	4.390	2.391	6.500
ridge	Autumn	Mid-level vegetation	3.565	0.392	6.464
ridge	Winter	Mid-level vegetation	5.730	0.813	10.222
ridge	All	Grass	-26.356	-29.248	-23.473
ridge	Spring	Grass	-30.197	-34.666	-25.818

ridge	Summer	Grass	-3.378	-6.505	-0.435
ridge	Autumn	Grass	-20.108	-25.860	-14.397
ridge	Winter	Grass	-30.480	-39.702	-21.466
lasso-ols	All	Greenspace	-3.631	-3.970	-3.295
lasso-ols	Spring	Greenspace	-2.378	-2.932	-1.770
lasso-ols	Summer	Greenspace	-1.281	-1.709	-0.851
lasso-ols	Autumn	Greenspace	-3.063	-3.750	-2.400
lasso-ols	Winter	Greenspace	-4.362	-5.342	-3.372
lasso-ols	All	Tree	-3.070	-3.519	-2.643
lasso-ols	Spring	Tree	0.377	-0.345	1.061
lasso-ols	Summer	Tree	-2.154	-2.738	-1.565
lasso-ols	Autumn	Tree	-2.949	-3.768	-2.072
lasso-ols	Winter	Tree	-4.397	-5.492	-3.269
lasso-ols	All	Mid-level vegetation	2.891	1.234	4.449
lasso-ols	Spring	Mid-level vegetation	-9.788	-12.568	-6.869
lasso-ols	Summer	Mid-level vegetation	4.343	2.304	6.277
lasso-ols	Autumn	Mid-level vegetation	3.296	0.158	6.309
lasso-ols	Winter	Mid-level vegetation	4.848	-0.034	9.502
lasso-ols	All	Grass	-26.544	-29.352	-23.768
lasso-ols	Spring	Grass	-30.026	-34.381	-25.608
lasso-ols	Summer	Grass	-3.713	-6.907	-0.606
lasso-ols	Autumn	Grass	-20.577	-26.103	-14.936
lasso-ols	Winter	Grass	-31.327	-40.112	-21.857
ridge-ols	All	Greenspace	-3.841	-4.177	-3.504
ridge-ols	Spring	Greenspace	-2.358	-2.992	-1.746
ridge-ols	Summer	Greenspace	-1.280	-1.709	-0.845
ridge-ols	Autumn	Greenspace	-3.054	-3.736	-2.398
ridge-ols	Winter	Greenspace	-4.384	-5.335	-3.409
ridge-ols	All	Tree	-3.328	-3.754	-2.884
ridge-ols	Spring	Tree	0.400	-0.318	1.096
ridge-ols	Summer	Tree	-2.157	-2.687	-1.632
ridge-ols	Autumn	Tree	-2.928	-3.848	-2.048
ridge-ols	Winter	Tree	-4.421	-5.578	-3.291
ridge-ols	All	Mid-level vegetation	2.779	1.262	4.553
ridge-ols	Spring	Mid-level vegetation	-9.748	-12.567	-6.861
ridge-ols	Summer	Mid-level vegetation	4.346	2.253	6.307

ridge-ols	Autumn	Mid-level vegetation	3.519	0.601	6.331
ridge-ols	Winter	Mid-level vegetation	4.995	0.119	9.770
ridge-ols	All	Grass	-26.390	-29.209	-23.647
ridge-ols	Spring	Grass	-30.241	-34.519	-26.147
ridge-ols	Summer	Grass	-3.613	-6.780	-0.408
ridge-ols	Autumn	Grass	-20.486	-25.932	-14.689
ridge-ols	Winter	Grass	-31.336	-39.782	-22.577
dt-ols	All	Greenspace	-0.705	-1.032	-0.389
dt-ols	Spring	Greenspace	-1.099	-1.559	-0.648
dt-ols	Summer	Greenspace	-0.144	-0.476	0.204
dt-ols	Autumn	Greenspace	-0.912	-1.443	-0.402
dt-ols	Winter	Greenspace	-0.652	-1.347	0.086
dt-ols	All	Tree	-0.186	-0.566	0.193
dt-ols	Spring	Tree	-0.453	-1.039	0.115
dt-ols	Summer	Tree	0.333	-0.087	0.750
dt-ols	Autumn	Tree	-0.320	-1.001	0.416
dt-ols	Winter	Tree	-0.697	-1.517	0.206
dt-ols	All	Mid-level vegetation	2.197	0.845	3.584
dt-ols	Spring	Mid-level vegetation	-1.937	-3.848	-0.107
dt-ols	Summer	Mid-level vegetation	0.895	-0.679	2.330
dt-ols	Autumn	Mid-level vegetation	0.910	-1.335	3.244
dt-ols	Winter	Mid-level vegetation	3.768	0.654	6.843
dt-ols	All	Grass	-5.582	-8.082	-3.292
dt-ols	Spring	Grass	-6.314	-8.955	-3.575
dt-ols	Summer	Grass	-1.117	-3.459	1.361
dt-ols	Autumn	Grass	-6.735	-10.980	-2.227
dt-ols	Winter	Grass	-0.503	-6.626	5.820
rf-ols	All	Greenspace	-0.973	-1.270	-0.675
rf-ols	Spring	Greenspace	-1.612	-2.124	-1.119
rf-ols	Summer	Greenspace	-0.055	-0.399	0.284
rf-ols	Autumn	Greenspace	-0.957	-1.480	-0.396
rf-ols	Winter	Greenspace	0.838	0.147	1.536
rf-ols	All	Tree	-0.433	-0.812	-0.018
rf-ols	Spring	Tree	-0.439	-1.047	0.188
rf-ols	Summer	Tree	0.570	0.149	0.971
rf-ols	Autumn	Tree	-0.465	-1.132	0.245

rf-ols	Winter	Tree	-0.171	-1.036	0.705
rf-ols	All	Mid-level vegetation	1.677	0.373	3.008
rf-ols	Spring	Mid-level vegetation	-3.486	-5.504	-1.663
rf-ols	Summer	Mid-level vegetation	1.810	0.537	3.119
rf-ols	Autumn	Mid-level vegetation	1.150	-1.042	3.328
rf-ols	Winter	Mid-level vegetation	7.972	4.464	11.557
rf-ols	All	Grass	-7.891	-10.199	-5.555
rf-ols	Spring	Grass	-11.649	-14.673	-8.774
rf-ols	Summer	Grass	-2.622	-4.997	-0.373
rf-ols	Autumn	Grass	-6.364	-10.656	-2.126
rf-ols	Winter	Grass	0.815	-4.965	6.573
gbdt-ols	All	Greenspace	-0.956	-1.234	-0.693
gbdt-ols	Spring	Greenspace	-1.007	-1.402	-0.599
gbdt-ols	Summer	Greenspace	0.104	-0.180	0.394
gbdt-ols	Autumn	Greenspace	-0.626	-1.077	-0.183
gbdt-ols	Winter	Greenspace	0.384	-0.232	1.004
gbdt-ols	All	Tree	-0.298	-0.628	0.056
gbdt-ols	Spring	Tree	-0.010	-0.478	0.476
gbdt-ols	Summer	Tree	0.458	0.087	0.824
gbdt-ols	Autumn	Tree	-0.474	-1.073	0.082
gbdt-ols	Winter	Tree	0.042	-0.703	0.772
gbdt-ols	All	Mid-level vegetation	-0.602	-1.493	0.347
gbdt-ols	Spring	Mid-level vegetation	-3.553	-4.846	-2.226
gbdt-ols	Summer	Mid-level vegetation	-0.037	-1.092	0.999
gbdt-ols	Autumn	Mid-level vegetation	0.079	-1.496	1.540
gbdt-ols	Winter	Mid-level vegetation	2.313	-0.159	4.676
gbdt-ols	All	Grass	-6.768	-8.370	-5.099
gbdt-ols	Spring	Grass	-7.672	-9.433	-5.832
gbdt-ols	Summer	Grass	-1.409	-3.032	0.277
gbdt-ols	Autumn	Grass	-3.317	-5.925	-0.785
gbdt-ols	Winter	Grass	-0.192	-4.390	4.135
lgbm-ols	All	Greenspace	-1.026	-1.340	-0.714
lgbm-ols	Spring	Greenspace	-1.466	-1.946	-1.006
lgbm-ols	Summer	Greenspace	0.345	0.019	0.689
lgbm-ols	Autumn	Greenspace	-0.473	-0.966	-0.012
lgbm-ols	Winter	Greenspace	0.691	0.078	1.307

lgbm-ols	All	Tree	-0.452	-0.887	-0.037
lgbm-ols	Spring	Tree	-0.191	-0.752	0.370
lgbm-ols	Summer	Tree	0.892	0.488	1.299
lgbm-ols	Autumn	Tree	-0.004	-0.611	0.600
lgbm-ols	Winter	Tree	0.372	-0.389	1.149
lgbm-ols	All	Mid-level vegetation	-0.354	-1.566	0.874
lgbm-ols	Spring	Mid-level vegetation	-2.748	-4.537	-0.876
lgbm-ols	Summer	Mid-level vegetation	0.274	-1.269	1.730
lgbm-ols	Autumn	Mid-level vegetation	0.642	-1.269	2.582
lgbm-ols	Winter	Mid-level vegetation	3.216	0.188	6.364
lgbm-ols	All	Grass	-6.041	-7.997	-4.063
lgbm-ols	Spring	Grass	-9.767	-12.405	-6.877
lgbm-ols	Summer	Grass	-2.315	-4.386	-0.218
lgbm-ols	Autumn	Grass	-5.686	-9.185	-2.327
lgbm-ols	Winter	Grass	1.770	-3.280	6.646

Table S3. The estimated mean β_0 and β_1 and their 90% confidence intervals of the equation: $Y_{residual} = \alpha + (\beta_0 + \beta_1 W)T_{residual} + \epsilon$ (i.e., the equation (3) in the main text), using the xgb-ols DML framework across seasons and different greenspace indicators. Here, xgb-ols means the first stage of DML applied the XGBoost model, while the second stage applied the ordinary least squares model.

Treatment	Season	β	mean	lower CI	upper CI
Greenspace	All	β_1	-0.377	-0.687	-0.074
Greenspace	All	β_0	-0.614	-0.883	-0.340
Tree	All	β_1	-0.212	-0.636	0.223
Tree	All	β_0	0.025	-0.365	0.405
Mid-level vegetation	All	β_1	-0.011	-1.368	1.397
Mid-level vegetation	All	β_0	-0.408	-1.589	0.879
Grass	All	β_1	4.067	1.575	6.350
Grass	All	β_0	-5.481	-7.472	-3.501
Greenspace	Spring	β_1	0.067	-0.540	0.616
Greenspace	Spring	β_0	-1.224	-1.669	-0.777
Tree	Spring	β_1	0.750	0.072	1.420
Tree	Spring	β_0	-0.194	-0.697	0.312
Mid-level vegetation	Spring	β_1	-0.743	-3.030	1.402
Mid-level vegetation	Spring	β_0	-2.264	-4.112	-0.316
Grass	Spring	β_1	-1.992	-5.532	1.557
Grass	Spring	β_0	-8.798	-11.587	-5.996
Greenspace	Summer	β_1	-0.331	-0.667	0.001
Greenspace	Summer	β_0	0.410	0.066	0.735
Tree	Summer	β_1	0.199	-0.266	0.625
Tree	Summer	β_0	0.747	0.333	1.158
Mid-level vegetation	Summer	β_1	-1.734	-3.287	-0.307
Mid-level vegetation	Summer	β_0	0.628	-0.766	2.072
Grass	Summer	β_1	1.519	-0.904	4.190
Grass	Summer	β_0	-1.352	-3.517	0.831
Greenspace	Autumn	β_1	-1.259	-1.837	-0.697
Greenspace	Autumn	β_0	-0.689	-1.193	-0.171
Tree	Autumn	β_1	-1.788	-2.480	-1.190
Tree	Autumn	β_0	-0.008	-0.621	0.597

Mid-level vegetation	Autumn	β_1	-0.547	-2.898	1.901
Mid-level vegetation	Autumn	β_0	0.168	-1.679	2.070
Grass	Autumn	β_1	3.941	-0.402	7.916
Grass	Autumn	β_0	-6.232	-9.767	-2.758
Greenspace	Winter	β_1	0.141	-0.486	0.769
Greenspace	Winter	β_0	0.403	-0.268	1.051
Tree	Winter	β_1	-0.116	-1.015	0.729
Tree	Winter	β_0	-0.003	-0.854	0.855
Mid-level vegetation	Winter	β_1	1.036	-2.185	4.144
Mid-level vegetation	Winter	β_0	3.532	0.567	6.599
Grass	Winter	β_1	8.889	2.777	15.900
Grass	Winter	β_0	2.089	-3.141	7.219

Table S4. The statistical results of the simple slope analysis for the treatment effects of greenspace on air pollution, calculated across different greenspace indicators and seasons, are presented as partitioned intervals. The "Interval code" column records the labels for different partitions; The "Significant?" column indicates whether the treatment effect within each partition is statistically significant ("Yes" for significant, "No" for not significant); The "Count" column records the number of streets included in each partition; The "Lower H/W ratio" column provides the minimum height-to-width ratio of streets within each partition; The "Upper H/W ratio" column provides the maximum height-to-width ratio of streets within each partition; The "Lower mean" column records the lowest average treatment effect within each partition; The "Upper mean" column records the highest average treatment effect within each partition.

Treatment	Season	Interval code	Significant?	Count	Lower H/W ratio	Upper H/W ratio	Lower mean	Upper mean
Greenspace	All	1	No	420	0.000	0.046	-0.336	-0.352
Greenspace	All	2	Yes	18083	0.046	9.948	-0.352	-3.879
Greenspace	Spring	1	Yes	17370	0.000	2.691	-1.273	-1.103
Greenspace	Spring	2	No	1133	2.693	9.948	-1.103	-0.646
Greenspace	Summer	1	Yes	11260	0.000	1.019	0.655	0.335
Greenspace	Summer	2	No	7243	1.019	9.948	0.335	-2.462
Greenspace	Autumn	1	No	7772	0.000	0.635	0.242	-0.514
Greenspace	Autumn	2	Yes	10731	0.635	9.948	-0.514	-11.603
Greenspace	Winter	1	No	18503	0.000	9.948	0.299	1.622
Tree	All	1	No	18503	0.000	9.948	0.182	-1.816
Tree	Spring	1	Yes	4385	0.000	0.332	-0.749	-0.513
Tree	Spring	2	No	14056	0.333	6.593	-0.513	3.929
Tree	Spring	3	Yes	62	6.682	9.948	3.992	6.310
Tree	Summer	1	Yes	17933	0.000	3.331	0.600	1.228
Tree	Summer	2	No	570	3.333	9.948	1.229	2.476
Tree	Autumn	1	Yes	5055	0.000	0.389	1.314	0.657
Tree	Autumn	2	No	7286	0.389	1.162	0.657	-0.651
Tree	Autumn	3	Yes	6162	1.163	9.948	-0.652	-15.512
Tree	Winter	1	No	18503	0.000	9.948	0.083	-1.011

Mid-level vegetation	All	1	No	18503	0.000	9.948	-0.399	-0.504
Mid-level vegetation	Spring	1	No	3974	0.000	0.294	-1.714	-1.920
Mid-level vegetation	Spring	2	Yes	10610	0.294	1.570	-1.921	-2.818
Mid-level vegetation	Spring	3	No	3919	1.570	9.948	-2.818	-8.711
Mid-level vegetation	Summer	1	Yes	2413	0.000	0.169	1.911	1.634
Mid-level vegetation	Summer	2	No	15894	0.169	4.535	1.634	-5.529
Mid-level vegetation	Summer	3	Yes	196	4.577	9.948	-5.598	-14.410
Mid-level vegetation	Autumn	1	No	18503	0.000	9.948	0.573	-4.579
Mid-level vegetation	Winter	1	No	5553	0.000	0.434	2.765	3.191
Mid-level vegetation	Winter	2	Yes	10936	0.435	2.170	3.191	4.893
Mid-level vegetation	Winter	3	No	2014	2.170	9.948	4.893	12.520
Grass	All	1	Yes	14269	0.000	1.489	-8.489	-2.759
Grass	All	2	No	4061	1.489	4.702	-2.758	9.601
Grass	All	3	Yes	173	4.720	9.948	9.671	29.788
Grass	Spring	1	Yes	18417	0.000	6.014	-7.325	-18.659
Grass	Spring	2	No	86	6.035	9.948	-18.699	-26.076
Grass	Summer	1	Yes	420	0.000	0.046	-2.475	-2.409
Grass	Summer	2	No	18083	0.046	9.948	-2.409	11.818
Grass	Autumn	1	Yes	13394	0.000	1.327	-9.147	-4.199
Grass	Autumn	2	No	5109	1.327	9.948	-4.197	27.948
Grass	Winter	1	No	14263	0.000	1.487	-4.485	8.022
Grass	Winter	2	Yes	4240	1.488	9.948	8.030	79.174

Table S5. The influence of BVOC emissions on the associations between greenspace and air pollution.

Variable	Mean	Lower CI	Upper CI	Significant?
**Greenspace × BVOC**	0.126	0.012	0.252	Yes
Greenspace	-0.180	-0.361	0.012	No
**Tree × BVOC**	0.216	0.029	0.401	Yes
Tree	0.055	-0.195	0.306	No
**Mid-level vegetation × BVOC**	0.074	0.025	0.120	Yes
Mid-level vegetation	-0.291	-0.436	-0.155	Yes
**Grass × BVOC**	-0.123	-0.287	0.019	No
Grass	-0.081	-0.268	0.109	No

References

(1) Cardozo, R. A.; Feinberg, A.; Tovar, A.; Vilcassim, M. J. R.; Shelley, D.; Elbel, B.; Kaplan, S.; Wyka, K.; Rule, A. M.; Gordon, T.; Thorpe, L. E. A Protocol for Measuring the Impact of a Smoke-Free Housing Policy on Indoor Tobacco Smoke Exposure. *BMC Public Health* **2019**, *19* (1), 666. https://doi.org/10.1186/s12889-019-7043-3.

(2) Ma, J.; Tao, Y.; Kwan, M.-P.; Chai, Y. Assessing Mobility-Based Real-Time Air Pollution Exposure in Space and Time Using Smart Sensors and GPS Trajectories in Beijing. *Ann. Am. Assoc. Geogr.* **2020**, *110* (2), 434–448. https://doi.org/10.1080/24694452.2019.1653752.

(3) Mazaheri, M.; Clifford, S.; Yeganeh, B.; Viana, M.; Rizza, V.; Flament, R.; Buonanno, G.; Morawska, L. Investigations into Factors Affecting Personal Exposure to Particles in Urban Microenvironments Using Low-Cost Sensors. *Environ. Int.* **2018**, *120*, 496–504. https://doi.org/10.1016/j.envint.2018.08.033.

(4) Huang, J.; Kwan, M.-P.; Cai, J.; Song, W.; Yu, C.; Kan, Z.; Yim, S. H.-L. Field Evaluation and Calibration of Low-Cost Air Pollution Sensors for Environmental Exposure Research. *Sensors* **2022**, *22* (6), 2381. https://doi.org/10.3390/s22062381.

(5) Michael, H.; Lim, C. C. *AirBeam2 Technical Specifications, Operation & Performance*. HabitatMap Environmental Tech & AirBeam. https://www.habitatmap.org/blog/airbeam2-technical-specifications-operation-performance (accessed 2025-08-14).

(6) Crilley, L. R.; Shaw, M.; Pound, R.; Kramer, L. J.; Price, R.; Young, S.; Lewis, A. C.; Pope, F. D. Evaluation of a Low-Cost Optical Particle Counter (Alphasense OPC-N2) for Ambient Air Monitoring. *Atmospheric Meas. Tech.* **2018**, *11* (2), 709–720. https://doi.org/10.5194/amt-11-709-2018.

(7) Manikonda, A.; Zíková, N.; Hopke, P. K.; Ferro, A. R. Laboratory Assessment of Low-Cost PM Monitors. *J. Aerosol Sci.* **2016**, *102*, 29–40. https://doi.org/10.1016/j.jaerosci.2016.08.010.

(8) Holstius, D. M.; Pillarisetti, A.; Smith, K. R.; Seto, E. Field Calibrations of a Low-Cost Aerosol Sensor at a Regulatory Monitoring Site in California. *Atmospheric Meas. Tech.* **2014**, *7* (4), 1121–1131. https://doi.org/10.5194/amt-7-1121-2014.

(9) Jayaratne, R.; Liu, X.; Thai, P.; Dunbabin, M.; Morawska, L. The Influence of Humidity on the Performance of a Low-Cost Air Particle Mass Sensor and the Effect of Atmospheric Fog. *Atmospheric Meas. Tech.* **2018**, *11* (8), 4883–4890. https://doi.org/10.5194/amt-11-4883-2018.

(10) *Optuna - A hyperparameter optimization framework*. Optuna. https://optuna.org/ (accessed 2025-10-28).

(11) *3.1. Cross-validation: evaluating estimator performance*. scikit-learn. https://scikit-learn/stable/modules/cross_validation.html (accessed 2025-10-28).

(12) Tolan, J.; Yang, H.-I.; Nosarzewski, B.; Couairon, G.; Vo, H. V.; Brandt, J.; Spore, J.; Majumdar, S.; Haziza, D.; Vamaraju, J.; Moutakanni, T.; Bojanowski, P.; Johns, T.; White, B.; Tiecke, T.; Couprie, C. Very High Resolution Canopy Height Maps from RGB Imagery Using Self-Supervised Vision Transformer and Convolutional Decoder Trained on Aerial Lidar. *Remote Sens. Environ.* **2024**, *300*, 113888. https://doi.org/10.1016/j.rse.2023.113888.

(13) Kan, Z.; Kwan, M.-P.; Cai, J.; Liu, Y.; Liu, D. Nonstationary Relationships among Individuals' Concurrent Exposures to Noise, Air Pollution and Greenspace: A Mobility-Based Study Using GPS and Mobile Sensing Data. *Health Place* **2023**, *83*, 103115. https://doi.org/10.1016/j.healthplace.2023.103115.

(14) Liu, D.; Kwan, M.-P.; Kan, Z.; Liu, Y. Examining Individual-Level Tri-Exposure to Greenspace and Air/Noise Pollution Using Individual-Level GPS-Based Real-Time Sensing Data. *Soc. Sci. Med.* **2023**, *329*, 116040. https://doi.org/10.1016/j.socscimed.2023.116040.

(15) Liu, Y.; Kwan, M.-P.; Yu, C. The Uncertain Geographic Context Problem (UGCoP) in Measuring People's Exposure to Green Space Using the Integrated 3S Approach. *Urban For. Urban Green.* **2023**, *85*, 127972. https://doi.org/10.1016/j.ufug.2023.127972.
(16) Skakun, S.; Wevers, J.; Brockmann, C.; Doxani, G.; Aleksandrov, M.; Batič, M.; Frantz, D.; Gascon, F.; Gómez-Chova, L.; Hagolle, O.; López-Puigdollers, D.; Louis, J.; Lubej, M.; Mateo-García, G.; Osman, J.; Peressutti, D.; Pflug, B.; Puc, J.; Richter, R.; Roger, J.-C.; Scaramuzza, P.; Vermote, E.; Vesel, N.; Zupanc, A.; Žust, L. Cloud Mask Intercomparison eXercise (CMIX): An Evaluation of Cloud Masking Algorithms for Landsat 8 and Sentinel-2. *Remote Sens. Environ.* **2022**, *274*, 112990. https://doi.org/10.1016/j.rse.2022.112990.
(17) Wang, J.; Kwan, M.-P.; Xiu, G.; Peng, X.; Liu, Y. Investigating the Neighborhood Effect Averaging Problem (NEAP) in Greenspace Exposure: A Study in Beijing. *Landsc. Urban Plan.* **2024**, *243*, 104970. https://doi.org/10.1016/j.landurbplan.2023.104970.
(18) *Traffic Data of Strategic / Major Roads - Traffic Speed, Volume and Road Occupancy (Raw Data) | DATA.GOV.HK*. https://data.gov.hk/en-data/dataset/hk-td-sm_4-traffic-data-strategic-major-roads/resource/5004b00d-aae3-4754-8eab-2ffdaf1e079e (accessed 2025-10-26).
(19) Zhu, L.; Zhang, Y.; Wang, F.; Chen, B.; Jin, L. N.; Dai, H.; Han, Y.; Lu, X.; Fu, T.; Gao, M. Undesirable Effects of Biogenic Emissions From Urban Green Spaces on Air Quality Are Counteracted by Their Transpiration and Dry Deposition. *Earths Future* **2025**, *13* (12), e2025EF006449. https://doi.org/10.1029/2025EF006449.
(20) Barwise, Y.; Kumar, P. Designing Vegetation Barriers for Urban Air Pollution Abatement: A Practical Review for Appropriate Plant Species Selection. *Npj Clim. Atmospheric Sci.* **2020**, *3* (1), 1–19. https://doi.org/10.1038/s41612-020-0115-3.
(21) EPD. *2022 Hong Kong Emission Inventory Report*; 2024. https://www.epd.gov.hk/epd/sites/default/files/epd/data/2022_Emission_Inventory_Report_Eng.pdf.
